# Community readiness for the program of all-inclusive care for the elderly (PACE): A qualitative study with Korean Americans in Los Angeles

**DOI:** 10.1371/journal.pone.0345750

**Published:** 2026-03-30

**Authors:** Juyoung Park, Seo-Yun Choi, Nan Sook Park, Jong Won Min, Tei Park, Yuri Jang

**Affiliations:** 1 Edward R. Roybal Institute on Aging, Suzanne Dworak-Peck School of Social Work, University of Southern California, Los Angeles, California, United States of America; 2 School of Social Work, University of South Florida, Tampa, California, United States of America; 3 School of Social Work, San Diego State University, San Diego, California, United States of America; 4 Hampshire College, Amherst, Massachusetts, United States of America; 5 Department of Social Welfare, Ewha Womans University, Seoul, Republic of Korea; Port Said University Faculty of Nursing, EGYPT

## Abstract

The Program of All-Inclusive Care for the Elderly (PACE), which provides integrated medical, social, and personal healthcare to help older adults remain in their homes and communities, has been a successful long-term care services and support (LTSS) model. Yet little is known about how racial and ethnic minority communities perceive and respond to this program. Guided by the Community Readiness Model (CRM), this explorative qualitative study examined readiness for PACE in Korean American communities in Los Angeles. Data were collected from 28 participants, including two focus groups with 19 older adults (age ≥ 55) and nine individual interviews with family caregivers and health and social service providers. Four major themes emerged: (1) Awareness, (2) Perceived Benefits, (3) Perceived Barriers and Concerns, and (4) Recommendations for Implementation and Outreach. In general, awareness of PACE was low among participants, and many had unclear or incorrect understanding of the program. Perceived benefits included the ability to age in place, access to coordinated care at a single setting, opportunities for social engagement, and reduced caregiving burden. Concerns noted by participants included the need to switch physicians, possible disruption of existing services such as In-Home Supportive Services, and uncertainty about the qualifications of PACE providers. Recommendations highlighted the importance of culturally tailored outreach, a more descriptive program name, strong leadership, a cooperative interdisciplinary team culture, and staffing that combines clinical expertise with compassion and cultural sensitivity. These findings suggest that introducing PACE in the Korean American community requires intentional adaptation to address both cultural expectations and structural barriers. This study offers insights into how the PACE model can be more effectively introduced, adapted, and sustained in racial and ethnic minority communities that have not been fully engaged by LTSS outreach and delivery efforts.

## Introduction

As the older adult population in the United States continues to grow, the demand for comprehensive long-term care solutions has increased. Long-term care services and supports (LTSS) are essential for helping older adults maintain their independence and quality of life, particularly in the context of chronic illness and functional limitations [1,2]. One innovative LTSS model is the Program of All-Inclusive Care for the Elderly (PACE), a capitated, government-funded program that primarily serves individuals dually eligible for Medicare and Medicaid [3]. Originating from the On Lok adult day health model established in 1973, PACE was formally authorized as a permanent Medicare provider and a state Medicaid option under the Balanced Budget Act of 1997 [3,4]. PACE delivers integrated medical, social, and personal care to older adults who require nursing home-level support but can remain safely in their homes and communities [3,5,6]. By coordinating care through interdisciplinary teams within a single organizational structure, PACE enables older adults to fulfill their desire to age in place [7–9].

A substantial body of literature demonstrates the effectiveness of PACE with participants experiencing lower hospitalization rates and delayed nursing home placement compared to non-participants [9–12]. Also, a high level of satisfaction with the program has been reported by both PACE participants and their family members [13]. Despite its demonstrated effectiveness, enrollment in PACE has remained disproportionately low among racial and ethnic minority populations [14]. This underrepresentation is particularly concerning given that PACE is designed to support individuals who are dually eligible for Medicare and Medicaid (commonly referred to as ‘Medi-Medi’ beneficiaries)—a population that often faces significant economic hardship and complex care needs. It is notable that nearly half of all dual-eligible individuals belong to a racial or ethnic minority group [15]. Contributing factors to this underutilization may include limited awareness of the program, outreach strategies that are not culturally and linguistically aligned with target communities, and a lack of partnerships between PACE organizations and community-based groups [14].

In the Los Angeles area, several PACE centers offer culturally and linguistically tailored care for Asian American communities. For example, Chinatown Service Center (CSC) PACE and HeritAGE PACE serve Asian Americans more broadly, while K-Day PACE specifically caters to Korean Americans by providing services delivered by Korean-speaking professionals. Koreans represent the fifth largest subgroup within the Asian American population, and a significant portion resides in the Los Angeles area [16]. Older Korean Americans often face health and social vulnerabilities, including language and cultural barriers, generational gaps and family disruptions, physical and mental health challenges, social isolation, and limited access to resources and services [17,18]. Despite the potential benefits PACE may offer, little is known about how the program is perceived and received within Korean American communities. Therefore, the aim of this present study is to conduct an exploratory qualitative assessment of awareness, needs, and community readiness for PACE among Korean Americans in Los Angeles.

This assessment is broadly guided by the Community Readiness Model (CRM) [19], which offers a structured framework for evaluating a community’s stage of readiness by examining both contextual factors and collective perceptions. The model outlines a continuum of readiness stages from ‘no awareness’ where the issue is not recognized, to ‘community ownership’ where the issue is fully supported and sustained by local leadership and resources [19]. In this study, CRM served as a conceptual lens rather than a formal staging tool, informing data collection and analytic interpretation without implying formal readiness stage classifications. Its application allows for the identification of local strengths, knowledge gaps, and opportunities to develop culturally responsive engagement strategies to support the adoption of PACE. Community readiness is explored through focus groups with older Korean Americans and qualitative interviews with key informants, including family caregivers and health and social service providers in Korean American communities.

## Methods

### Qualitative data collection

Participants were recruited through the research team’s existing community networks and referrals from Korean-serving healthcare clinics, which facilitated access to older adults who were willing and able to participate in group discussions and provide informed consent. Eligibility criteria for the focus groups included being a community-dwelling Korean American aged 55 or older, residing in the Los Angeles area, and being able to understand study procedures and provide informed consent. Individual interviews were conducted with Korean Americans aged 18 or older who provide care for aging family members, as well as with health and social service providers who work closely with older Korean Americans in the Korean American communities of Los Angeles. Informed consent was obtained from all participants prior to data collection, and the consent procedure was approved by the Institutional Review Board of the University of Southern California.

Focus groups were chosen as the preferred method for engaging older adults, as they provide a shared setting in which participants can collectively reflect on their experiences. This approach facilitates rich dialogue, peer validation, and the emergence of both common and divergent perspectives. In contrast, individual interviews were deemed more appropriate for caregivers and professionals, as they offer the logistical flexibility needed to accommodate caregiving responsibilities and demanding work schedules. This format also allows for more in-depth and confidential insights into caregiving dynamics and service delivery contexts.

Focus groups were hosted on-site in a local clinic’s conference room, while individual interviews were conducted online via Zoom. Focus group discussions lasted approximately 90 minutes, and individual interviews about 60 minutes. Both focus groups and interviews began with a brief background questionnaire covering demographic characteristics and health status. For caregivers and professionals, the questionnaire also included questions about years of caregiving experience or years of service in their respective fields.

Conversations were guided by semi-structured questions. For example, older adult focus group participants were asked, “Have you heard of the PACE program?”; “What difficulties have you experienced accessing health care services?”; and “How would you feel about having a comprehensive, all-in-one health care service in your neighborhood?”. Exemplary interview questions for caregivers and professionals include, “What has been your experience supporting older adults in accessing services?”; “How do you see the potential fit of the PACE model within the Korean American community?”; and “What factors do you think would support or challenge the implementation of PACE in this community?”. All discussions were conducted in Korean, and audio-recorded with participants’ consent. Participants received gift cards valued at $50 and $100 for individual interviews. The study received approval from the university’s Institutional Review Board (IRB).

During May and June of 2025, a total of 28 individuals participated in the study. Written informed consent was obtained from all participants. Nineteen older adults participated in two focus groups (one with eight participants and the other with eleven). Individual interviews were conducted with two family caregivers and seven professionals, including a social worker, senior center administrator, senior housing coordinator, and nurse practitioner.

### Analytic Strategy

Audio recordings were transcribed verbatim in Korean. Using the constant comparison method [20], each transcript was independently coded by the research team. Individual codes and interpretations were compared and discussed, refining and modifying them throughout the process. Coding began with open coding, after which codes were refined and grouped into broader categories to identify emerging themes [21].

To ensure the trustworthiness and rigor of the study, we employed strategies such as prolonged engagement with the data, triangulation, and detailed record keeping [22]. Triangulation was achieved by drawing on multiple data sources, including focus groups with older adults and interviews with caregivers and professionals, to compare and corroborate findings and by conducting multiple rounds of cross-coding to incorporate diverse perspectives. We also maintained analytic memos, including process recordings and reflections on codes and discrepancies. These records informed ongoing coding decisions and thematic interpretation.

Three authors (JP, SC, and YJ), who also participated in the interviews, read the transcripts in Korean and immersed themselves in the data. Each independently developed a preliminary code list in English. Using these lists, the coders conducted line-by-line coding of the first two interviews, with the flexibility to add new codes as needed. They then compared their coded transcripts, discussed any discrepancies, and recoded the interviews using a set of consensus codes.

The same process was repeated with two additional transcripts. Through this iterative approach, the team reached consensus that the coding could be reliably applied across transcripts using the finalized codebook. Two coders (JP and SC) then coded the remaining transcripts using a consensus approach, coding independently and resolving disagreements through discussion. The coded transcripts were reviewed by another researcher (NP), adding an additional layer of reliability and helping to resolve any discrepancies. We used the qualitative data analysis software Atlas.ti (Scientific Software Development, Berlin) to manage and analyze the data. As a final step, selected quotes were translated into English to preserve the original meaning and nuance of the participants’ statements [23].

## Results

The two focus groups included 19 older Korean American adults, with a mean age of 72 years (*SD* = 9.8; range, 55–84 years). Twelve were women (63%), and all participants were born in Korea and used Korean as their primary language. Participants had lived in the U.S. for an average of 33.6 years (*SD* = 5.9; range, 20–45 years). The two caregivers were women born in Korea who had immigrated to the U.S. 42 and 15 years ago, respectively. One provided care for her 89-year-old mother, and the other was caring for her 77-year-old mother-in-law who had been diagnosed with dementia. Seven professionals were interviewed, including three social workers, two senior center administrators, a senior housing coordinator, and a nurse practitioner. All had extensive experience working with older Korean American adults and were currently employed in community-based settings in Los Angeles. Of the seven participants, six were women (86%). All professionals were in their 50s and had an average of 12.1 years of work experience (*SD* = 7.0; range, 2–23 years).

The qualitative analysis of focus group discussions and interviews revealed a wide range of perceptions and various perspectives regarding PACE. Findings are organized into four overarching themes: (1) Awareness, (2) Perceived Benefits, (3) Perceived Barriers and Concerns, and (4) Recommendations for Outreach and Implementation. These themes represent various stages of community readiness and reflect both contextual and perceptual dynamics emphasized in the Community Readiness Model [19], as well as cultural characteristics unique to Korean Americans. Table 1 summarizes the themes and subthemes that emerged from the qualitative data analyses. These themes reflect perspectives shared across participant groups, while acknowledging that certain subthemes were more salient within particular stakeholder contexts. Each theme is further elaborated in the text with exemplary participants quotes.

**Table 1:** Themes and Subthemes Emerged.

Theme	Subthemes
Awareness	Unawareness Unclear understanding Incorrect understanding
Perceived benefits	Aging in place with dignity Comprehensive and coordinated care Social engagement and mental stimulation Reduced caregiver burden
Perceived barriers and concerns	Physician switching requirement Trade-offs with In-Home Supportive Services (IHSS) Trust and qualifications of PACE providers Overlap and competition with existing services
Recommendations for outreach and implementation	Clarifying PACE’s unique value and branding Leadership and team culture Staffing for compassionate and culturally responsive care

### 1. Awareness

Despite generally low levels of knowledge about PACE among participants, a range of awareness and understanding was evident across the groups.

#### Unawareness.

Nearly all older adult and caregiver participants had never heard of PACE, indicating a complete lack of exposure and no awareness of the program’s existence. Among professionals, many were aware of the program, but their knowledge was limited. They noted that their understanding was based on brief encounters with information at professional conferences or through peer networks.

#### Unclear understanding.

Many of those who had heard of PACE did not have a clear understanding of what the program entailed. Their knowledge was often vague and partial. One social worker shared how the program’s name contributed to her initial misunderstanding:

“The program was introduced as ‘community-based services,’ which made me assume it was just one of many services already available. There are already so many things labeled ‘community-based.’ PACE seemed like just another program. So, I didn’t really think about it much at first. But later, I had a chance to work more closely with people from PACE and realized that it’s something much more comprehensive. That’s when I started looking into it more seriously.”

Naming confusion was more frequently observed among older adults, many of whom often conflated PACE with Adult Day Health Care (ADHC) or local clinics. One older Korean adult noted, “I don’t think the name itself ‘PACE’ carries any meaning for us as Koreans. It doesn’t click.” This lack of clarity led to many questions about the program’s purpose, eligibility criteria, and benefits.

#### Incorrect understanding.

Beyond limited exposure, early impressions of PACE were often shaped by assumptions rooted in incomplete information. Several participants initially believed PACE was a high-cost, private-pay program. Descriptions of it as an all-inclusive model led some to perceive it as a luxury service. One older adult shared:

“What PACE offers sounded like one of those luxury services—something you’d expect to see in a glossy brochure, not something actually accessible to people like us. It just sounded too ideal—almost like a dream. Is it really available to the public?”

These assumptions created a sense of psychological distance and skepticism, discouraging engagement even before the program was properly introduced. Professionals echoed this sentiment, noting that the lack of accessible information made it difficult to explain the program accurately or confidently refer older adults to PACE.

### 2. Perceived benefits

Once introduced to the concept of PACE, most participants responded positively, especially to elements of the model that addressed gaps in care delivery and unmet needs within their communities.

#### Aging in place with dignity.

The desire to remain at home emerged as a consistent and culturally salient value across all groups. Older adults expressed a strong preference for aging in place, viewing institutional care as a last resort. One participant stated, “Most people want to stay in their own homes if possible until the end. Going to an institution feels like being sent away.” Professionals echoed this sentiment, emphasizing that community-based care models align with the preferences of Korean American older adults and reflect deeply held, family-centered cultural values.

This theme was especially pronounced in the Los Angeles Koreatown context, where many older Korean Americans live apart from their adult children. In contrast to other areas where English language barriers may require older adults to live closer to family for daily support, Koreatown’s high concentration of Korean-language services enables older adults to maintain independence while navigating daily life in their native language. As one community leader at a senior center explained, “For those living apart from their children, being able to receive expert care in Korean is incredibly important.” He strongly endorsed PACE’s mission to help older adults remain in the homes and communities where they feel most comfortable.

#### Comprehensive and coordinated care.

The integrated service delivery model offered by PACE, encompassing medical, social, rehabilitative, and nutritional support, was highly endorsed by older adults, caregivers, and professionals. Participants appreciate the appeal of a “one-stop” system that could eliminate the need to navigate multiple providers and fragmented services. As one focus group participant shared: “It would be great to have everything arranged in one place.”

Professionals noted that this model is especially beneficial for clients with multiple chronic conditions, who often struggle with siloed care systems. Caregivers echoed this sentiment from a logistical perspective. One caregiver explained, “What appealed to me was the idea of total care, which has everything in one place: nutritious meals, health services, and activities.” A social service provider also emphasized that integrated care programs, such as PACE, can support early detection of health issues and more effective management of comorbid conditions.

#### Social engagement and mental stimulation.

Focus group participants noted that older Korean Americans often experience social isolation, especially when their mobility is limited or when they spend long periods alone at home. The prospect of regular social interaction through culturally familiar group activities and group meals was viewed as both emotionally uplifting and cognitively stimulating. As one participant shared, “For people who can go out, simply being around others is a blessing.”

Professionals also echoed these sentiments, highlighting the elevated risk of depression and dementia among older adults who are mobility-challenged and socially isolated. They strongly endorsed structured group activities as meaningful non-medical interventions that promote social engagement and mental well-being.

#### Reduced caregiver burden.

While caregiver burden was most explicitly expressed by caregivers themselves, professionals also acknowledge the potential of PACE to alleviate family strains, particularly in dual-income households or among adult children juggling work and caregiving responsibilities. Caregivers frequently described the physical, emotional, and logistical challenges of providing consistent care with limited support. One relied on In-Home Supportive Services (IHSS), while another managed caregiving mostly on her own or with minimal support, often at the expense of her own health, career, or relationships.

The PACE model was seen as especially promising for Korean American families navigating the tension between cultural expectations of filial responsibility and the practical demands of daily life. As one caregiver explained:

“If PACE offers services that go beyond IHSS, I’d go for it. As a daughter, I am obliged to find the best way to keep my mother healthy and happy. It will also give me a chance to breathe and feel relieved, knowing she is well cared for.”

### 3. Perceived barriers and concerns

While participants were enthusiastic about various aspects of the PACE model, they also voiced concerns, particularly about systemic structures and cultural alignment, which could impact their trust in or willingness to participate in the program.

#### Physician switching requirement.

The requirement to switch primary care providers emerged as a significant concern across focus groups and individual interviews. Older Korean American participants often expressed deep trust in their current doctors, relationships built over many years of consistent care. One participant said, “My doctor knows everything about me,” reflecting the emotional comfort and clinical continuity tied to these long-standing connections.

Professionals affirmed this barrier, nothing that many older Korean Americans view their doctors not merely as medical providers, but as long-term partners in managing their health. “Older people feel comfortable with their doctor who knows their long life and health history. Changing feels like starting over,” a social worker explained. Even when PACE was presented as a comprehensive and coordinated model, participants were hesitant to risk losing a trusted relationship. Some professionals also noted that switching doctors could raise concerns about communication, further complicating the transition.

#### Tradeoffs with in-home supportive services (IHSS).

For family caregivers, the potential loss of IHSS hours was identified as a major barrier to enrolling in PACE. Caregivers described IHSS not only as essential for hands-on caregiving support but also as a stabilizing factor that enabled them to balance work, parenting, and other responsibilities. As one caregiver reflected, “IHSS has been what’s kept us going. We’ve had it for years. It’s the only way I’ve been able to keep working and still care for my mother.”

As such, any program that required giving up IHSS, even temporarily, was viewed with caution and, at times, resistance. “If it’s only 10 hours instead of the 180 hours we get now, I can’t do it,” one caregiver explained, underscoring concerns that PACE might not offer the same volume or flexibility of support currently provided through IHSS.

This concern was less salient among older adults, particularly those who were not currently receiving IHSS and therefore less aware of what might be lost. However, professionals recognized the implications of this tradeoff and acknowledged that it could significantly impact enrollment decisions. Some participants emphasized the importance of clear communication about the level of support PACE can provide to secure caregiver buy-in. Others stressed the need for policy coordination or transitional support strategies to ease the perceived burden of switching programs.

#### Trust and qualifications of PACE providers.

Concerns about transitioning into PACE extended beyond logistics to deeper questions of trust and provider quality. Many older adults expressed uncertainty about the qualifications of PACE staff and what would happen if the care provided did not meet their expectations. One focus group member asked, “What if I prefer my previous doctor? Can I go back?” This question reflected broader concerns about whether enrolling in PACE would limit their options or flexibility in care.

Participants also voiced their confusion about the process of care transition, highlighting the need for transparent communication about enrollment, exit options, and how continuity of care would be supported throughout the PACE. Participants also wondered how they could assess provider credibility in advance. Confidence in provider quality emerged as essential to participants’ openness to PACE.

#### Overlap and competition with existing services.

Some professionals expressed concerns that PACE may be perceived as redundant or in competition with existing community-based programs, such as ADHC centers or local clinics. “If it’s not very different from the ADHC, it may just end up shifting clients from one place to another,” one social worker remarked. This skepticism appeared to reflect a broader lack of clarity about how PACE differs in scope, coordination, and comprehensiveness. Without a clear understanding of its diverse features, such as interdisciplinary care teams, integrated service delivery, and in-home medical support, PACE risked being viewed as merely a repackaged version of services already available in the community. However, some professionals urged a shift in perspective—away from territorial concerns and toward shared goals. As one interviewee explained: “It’s like opening a restaurant near another. People naturally get territorial, as with any business model. But if we shift the focus to what’s truly best for older adults, there’s room for cooperation rather than competition among service providers.” This insight underscores the importance of centering older adults’ social and healthcare needs in implementation efforts and prioritizing partnerships over competition among existing providers. It also suggests that clearly articulating PACE’s distinct contribution may help foster a more collaborative ecosystem of care. Indeed, one senior center administrator welcomed PACE, noting that its services would be particularly well-suited for older adults with higher levels of frailty, whose needs exceed what the senior center is equipped to provide.

### 4. Recommendations for outreach and implementation

Participants offered concrete and culturally grounded recommendations to enhance awareness, understanding, and community uptake of PACE. These suggestions addressed not only informational gaps, but also deeper concerns about trust, cultural relevance, and the Korean American community’s readiness to engage with the program.

#### Clarifying PACE’s unique value and branding.

Participants emphasized the importance of clearly distinguishing PACE from commonly used services such as ADHC programs. Many participants initially perceived PACE as potentially duplicative until its broader scope was clarified. As one professional advised, “Promote it as a comprehensive program for those with more serious or complex healthcare needs”. The term ‘all-inclusive care’ resonated more once participants understood the full range of medical, social, and home-based service offered by PACE.

It is notable that there was broad consensus that the program’s branding, particularly the acronym “PACE”, lacked cultural relevance and linguistic clarity in Korean. Participants recommended using more descriptive and translatable language in outreach materials and advertisements. As one participant noted, “It should sound like something our elders can immediately understand.”

#### Leadership and team culture.

Several professionals emphasized that successful implementation of PACE would depend not only on the program’s design but also on the quality of its leadership. In particular, the need for visionary leadership capable of delegation was frequently highlighted. As one community leader stated:

“Any organization can fail if the leadership isn’t clear and compassionate. You need someone who sees the big picture and builds a team around that.”

She also noted that as the number of enrollees grows, so will the size and complexity of the professional care team, making respect and harmony within the team culture even more vital. A nurse practitioner echoed this sentiment, underscoring the importance of collaborative, interdisciplinary care:

“There won’t be any members or discipline teams that are less or more important to patients. Every single staff member has a role in improving health outcomes and supporting the mental well-being of older adults at PACE.”

This emphasis on shared responsibility and mutual respect highlights the importance of cultivating an environment where all staff feel valued and empowered to provide holistic person-centered care.

#### Staffing for compassionate and culturally responsive care.

While participants appreciated the availability of Korean-speaking staff in some existing PACE programs, they stressed that language alone was not sufficient. They called for well-trained professional teams who are not only linguistically and clinically skilled but also culturally attuned to Korean norms, values, and intergenerational caregiving dynamics. “We need teams who are truly skilled and compassionate,” said a nurse practitioner, emphasizing the importance of empathy and cultural sensitivity alongside medical and linguistic expertise.

Other participants echoed the need to recruit interprofessional team members who genuinely care for older adults, highlighting that emotional warmth can be as critical as technical competence. As one community leader reflected:

“I’ve seen doctors who are technically skilled but emotionally distant. But older people sense that. They may not say it out loud, but they can tell when someone is just doing a job versus when someone truly cares. You need people who work from the heart—who treat them with warmth, not just skills. If older adults feel they are genuinely treated at PACE, good words will spread out. There will be no need for promotional efforts.”

Participants also emphasized the importance of continuity in staffing and emotional attentiveness, both of which they equated with trustworthiness in care. Training efforts that extend beyond clinical competence to include humility and relational skill were viewed as essential for fostering long-term engagement. Several professionals also underscored the need for staff retention, noting that high turnover would undermine trust and continuity for PACE participants.

## Discussion

Despite the well-documented effectiveness of the Program of All-Inclusive Care for the Elderly (PACE) in improving health outcomes and supporting aging in place among older Americans [9–12], little is known about how racial and ethnic minority communities perceive or are prepared to engage with this model [14]. Guided by the Community Readiness Model (CRM) [19], this qualitative study explored the preparedness of the Korean American communities in Los Angeles, home to the largest Korean population in the U.S., to engage with PACE. Through focus groups with older adults and individual interviews with caregivers and service providers, we identified four key themes: (1) Awareness, (2) Perceived Benefits, (3) Perceived Barriers and Concerns, and (4) Recommendations for Outreach and Implementation. These findings shed light on both the opportunities and challenges of implementing PACE in this community, offering insights to guide culturally responsive expansion efforts.

Awareness of PACE among older Korean Americans, caregivers, and service providers was notably limited, with many participants confusing the program with Adult Day Health Care (ADHC) centers. This lack of recognition reflects how longstanding care models, including PACE, may remain unfamiliar in specific immigrant communities when culturally and linguistically tailored outreach has not been implemented. Notably, limited awareness was not only a matter of exposure but also of interpretation. Several participants initially assumed that PACE was a high-cost, private-pay service, highlighting how incomplete or unclear information can foster misconceptions and psychological distance. These assumptions align with broader findings that, when health programs are introduced without cultural and linguistic grounding, they may be perceived as inaccessible or irrelevant [14]. However, once accurate information was provided, participants’ perceptions shifted markedly. Many expressed enthusiasms for a model that could provide comprehensive and affordable care. This transformation underscores the critical role of culturally informed outreach and educational efforts in promoting program awareness and facilitating referrals within the community.

Perceived benefits of PACE emerged as the second major theme, illustrating how older Korean Americans, family caregivers, and service providers envisioned the program’s value. A primary appeal was the opportunity to age in place, a widely shared goal among older Americans [24]. Among older Korean Americans, this preference was deeply connected to cultural values of respect, dignity, and the desire to grow old in familiar environments.

Living in or near ethnic communities, where language, food, and social norms are culturally aligned, was seen as essential to maintaining identity, particularly for those with limited English proficiency or weakened social ties. Within this context, PACE’s integrated model that delivers medical, social, and personal care through a single coordinated system was viewed as particularly attractive. Prior research has shown that this type of integration is central to PACE’s effectiveness in reducing fragmented care and promoting continuity for older adults with multiple chronic conditions [5,11,25]. Participants believed these benefits were not only desirable but also feasible for meeting the needs of older Korean Americans.

Participants also emphasized the value of social and mental engagement opportunities, including shared meals and group activities, which they saw as essential for addressing loneliness and supporting emotional well-being [3]. Given the elevated health risks among older Korean Americans due to social and linguistic isolation [26], this aspect of the program was highly endorsed, particularly by service providers, as both a preventive and supportive measure. In addition, family caregivers viewed PACE as a potential source of relief from the demands of caregiving, especially for those balancing employment and intergenerational caregiving responsibilities. The prospect of shared responsibility through PACE was welcomed as a way to reduce both physical and emotional strain, while ensuring that loved ones receive high-quality, culturally attuned care.

Regarding perceived barriers, findings revealed how older Korean Americans and their families perceived new models of care through the lens of trust and familiarity. Many participants expressed reluctance to leave long-standing physicians, relationships that provided not only clinical continuity but also emotional reassurance in navigating the healthcare system. This hesitation extended to uncertainty about the qualifications of PACE providers and whether the quality of care would meet expectations. Family caregivers also emphasized their reliance on In-Home Support Services (IHSS), often describing it as the foundational support that made caregiving feasible. The possibility of losing IHSS hours to enroll in PACE was perceived as destabilizing, even when participants acknowledged potential benefits of a more integrated care model.

Professionals further noted that PACE might be perceived as overlapping with existing community-based programs such as ADHC centers, which have long been accessible and trusted in Los Angeles’s Korean American community. These concerns suggest that resistance to PACE stems less from skepticism about the program itself and more from fears of disrupting the established care systems families currently depend on. To support successful implementation, PACE should be introduced not as a duplication or replacement but as a complementary program, designed to work in partnerships with trusted providers and community services, while minimizing disruptions to caregiving routines and existing benefits.

Several participant recommendations highlighted key elements that could enhance the success of PACE implementation within the Korean American community. One major concern was the perception of the acronym “PACE” as abstract and culturally disconnected. This underscores the importance of naming and messaging that resonate with older adults and their families. For immigrant communities, unfamiliar program names can create a sense of distance, reinforcing the impression that the service is not for them.

Consistent with previous research on PACE, participants emphasized the need for linguistically accessible and culturally tailored outreach to build trust and relevance [14]. Beyond naming, participants underscored the importance of relational qualities in care delivery, including compassion, cultural humility, and consistency. These expectations extended to leadership, where participants stressed the value of a clear vision, team-based coordination, and a culture grounded in respect and collaboration. This emphasis aligns with findings from the integrated care literature, which show that successful implementation depends not only on program design, but also on strong leadership and an organizational culture that fosters interdisciplinary collaboration [27].

Taken together, these recommendations suggest that introducing PACE to older Korean Americans requires meaningful alignment with their cultural and community contexts. To succeed, the program should be embedded within trusted community spaces, staffed by professionals who reflect the community’s expectations, and led by organizations committed to equity, inclusion, and cultural respect. These strategies are essential for initial program uptake and sustaining long-term engagement and impact.

Several limitations should be considered when interpreting findings from this exploratory qualitative study. First, while efforts were made to include diverse perspectives, the number of participants in focus group discussions and individual interviews was limited. The two focus groups with older adults offered valuable group interaction; however, conducting a greater number of smaller-sized focus groups may have facilitated deeper dialogue and broader representation of views. Additionally, the inclusion of only two caregivers and one health care provider interview limit the comprehensiveness of subgroup-specific perspectives, particularly regarding caregiving experiences and clinical service provision. Second, the group setting of the focus group discussions may have introduced social desirability bias or inhibited participants from fully expressing their views due to group dynamics or cultural norms around deference and harmony. In addition, variability in health status, cognitive functioning, and prior service experiences among older adult participants may have influenced group dynamics and the depth of individual contributions. Third, the community-specific nature of this study, focusing on the Korean American community in Los Angeles, limits the generalizability of the findings to other populations or geographic areas. Future research should aim for broader and more balanced recruitment across stakeholder groups and may benefit from mixed method designs to strengthen validity and depth. Longitudinal studies that track changes in community awareness, perceptions, and implementation outcomes over time are also recommended.

Despite these limitations, the present study offers important insights into the interpersonal, structural, and cultural factors that shape community readiness for PACE among older Korean Americans. By identifying factors such as limited awareness, cultural perceptions of care, trust in existing providers, and preferences for culturally responsive care, this study contributes to a deeper understanding of how the PACE model can be more effectively introduced, adapted, and sustained within immigrant communities.

## Supporting information

S1 File English transcripts (de-identified). (PDF)
