# Supplementary material for: Community readiness for the program of all-inclusive care for the elderly (PACE): A qualitative study with Korean Americans in Los Angeles
Source: PLoS One. 2026 Mar 30;21(3):e0345750. doi: 10.1371/journal.pone.0345750 (PMC13035145; doi:10.1371/journal.pone.0345750)
Supplement: S1 File — (PDF) [file pone.0345750.s001.pdf]

S1 File. De-identified English Interview Transcripts

De-identified English-language transcripts of qualitative interviews conducted for the study "Community Readiness for the Program of All-Inclusive Care for the Elderly (PACE): A Qualitative Study with Korean Americans in Los Angeles."

Professional 1. Social Worker
[Portion omitted]

Have you heard of the PACE program?

PACE program… I'm sorry, but could you explain it in a bit more detail so I can make sure it matches what I know? I have not heard of it directly under that name. I vaguely heard that someone was planning to operate a program like that. I remember hearing that a physician in the Korean community was trying to start something related to it, but I am not very well connected to community rumors, so I do not know the details.

Among ourselves, we sometimes joke that older adults do so well at ADHC that even though there are many primary care physicians in Koreatown, they do not need to visit hospitals as often. In a way, that is a good thing. It suggests their health is being managed well. We even joke that "business must not be good," meaning that seniors are well cared for at the center.

Currently, our center does not have a physician on site. I have heard that some ADHCs have an Oriental medicine doctor on site, but I do not know where exactly. As you mentioned, I may have heard that there were plans to operate a program in collaboration with a physician who would be physically present. However, I do not know the exact details.

Most existing ADHC centers already operate within a fairly established system. So a model with a full-time on-site physician may still be in an early stage. If there were a PACE-like system with a physician physically present, I believe older adults would feel much more secure. In ADHC, nurses check blood pressure and monitor health daily. However, having a physician directly on site could create a different level of reassurance.

Older adults often feel secure even from seemingly small symbolic gestures. At a previous center where I worked, we always held their hands when seeing them off to prevent falls and personally assisted them when boarding the bus. We even purchased parking attendant vests for staff to wear while escorting them. Although it was somewhat inconvenient for us, the seniors really appreciated it. They said it made them feel protected in an organized way. Even symbolic elements like that can create a strong sense of safety.

If there were a system where they could see a physician at any time, I think they would feel even more reassured. However, I do not know how that would interact with their personal primary care physicians or how it would connect to the existing medical system. Seniors are very sensitive to information. If they hear that "that place is good," some will actually move. So if such a system were to become stable and trusted, I imagine some might transfer there. But this is only my personal speculation; I do not know the system precisely.

You mentioned that PACE has been around for some time. How might it overlap with ADHC?

From what I understand, PACE seems to have somewhat different priorities and goals compared to ADHC. In our case, if someone's health is too poor, or conversely too good, it becomes

difficult to provide services. Fundamentally, we are a center-based service, not a home-based intervention system.

We do provide temporary support in limited cases, such as if a participant is injured and unable to attend. We might provide meals or check in by phone, but that assumes they will return to the center. Home-visit–centered services are realistically difficult to operate and somewhat outside our current system.

If we determine that a participant's condition is too severe to manage in ADHC, we refer them elsewhere. To be honest, most often that means preparing for placement in a long-term care facility after consulting with family. However, such cases are not common and tend to involve very serious conditions, such as advanced dementia with significant safety risks. Otherwise, we try to accommodate most individuals.

It makes me wonder whether PACE may focus more on physical health management. In contrast, what older adults appreciate most about ADHC is the opportunity to meet people and participate in activities. Mental health support is important, but what seniors most often express gratitude for is surprisingly simple: reading letters to them, helping with forms, or assisting with applications for programs. Questions such as "What does this mean?" or "How do I apply?" are daily, practical issues that feel very meaningful to them.

Children are often busy or relationships may not be smooth, so this kind of social support becomes even more essential. From a social worker's perspective, these tasks may not be the top priority among all responsibilities. But for older adults, they often feel like the most important help. Personally, I receive the most gratitude when assisting with these matters.

Through hobbies and social interactions here, I can see seniors smiling at least once a day. They exercise together in ways they could not manage alone. That is why I sometimes describe ADHC as similar to a school. Getting up at a set time each day, dressing nicely, and coming to the center itself has meaning. Some come dressed very elegantly, as though it is their one opportunity to care for and express themselves.

When I ask, "If you do not come to the center, what do you do at home?" most say, "I just lie down," or "I watch YouTube." Without the center, many would lie down all day with no visitors. In that sense, ADHC is more focused on fostering social relationships.

Of course, as a senior health center, health management is the official goal. But in reality, the help seniors feel most strongly often comes from simple, everyday interactions.

Many participants appear relatively healthy on the surface, but most have multiple chronic conditions such as hypertension, diabetes, back pain, or mobility limitations. Some use walkers and move with difficulty. Even if we call them participants, in reality they are all patients in some way. Diagnosis and functional ability do not always match. Some have a dementia diagnosis but score perfectly on cognitive screening tests. Insurance criteria have become stricter, so those who appear healthy may not receive many approved service hours, even if they wish to attend daily.

Each center has different characteristics. Some serve relatively younger, active seniors; others have much older individuals with more severe conditions. In extreme cases, such as severe dementia with major safety risks, we coordinate with families for more appropriate placement. But most individuals, even if they appear stable, carry significant medical and functional challenges.

If you were to work within a PACE interdisciplinary team, are there any concerns about communication or collaboration?

If I were to work as a social worker within PACE, one concern would be communication with physicians. Sometimes, even when we document observations thoroughly, it can feel as though our reports are not fully acknowledged. For example, we may observe significant cognitive decline, but a physician may not see it the same way. At times, we do not receive clear feedback. I have wondered whether this reflects the positional hierarchy of social workers.

Of course, we do not make claims without basis. We report concerns based on observation and counseling, recognizing that final diagnosis is the physician's role. If collaboration within PACE were more immediate and mutually respectful, it would allow social workers to share observations efficiently and receive feedback, which would feel more stable and effective.

Another issue is counseling. In ADHC, an LCSW conducts formal counseling perhaps once every six months, but it is difficult to consider that sustained therapeutic intervention. If PACE had on-site counseling staff providing continuous mental health services, it could offer significant reassurance. Immediate access when needed would be beneficial.

I do not know, however, how PACE integrates non-medical needs such as hobbies or social activities. If it includes those elements as well, it could become a very meaningful model.

From your perspective, what areas could be improved?

I believe the existence of any structured system for older adults, whether PACE or ADHC, is itself meaningful. Before working in this field, I honestly felt uncomfortable around older adults. That changed completely through practice. I developed respect and began to understand the weight of their lives.

Older adults are constantly losing something, including health, people, relationships, and parts of their lives. When these losses are not fully processed, emotional difficulties can follow. Through this work, I have realized that simply having a place to go is profoundly important. Many seniors say, "When I come here, I feel welcomed," or "I feel treated with respect." Some even say, "No one wants to talk to me because I smell. Who would want to talk to me?" We cannot solve every problem, but simply listening can bring comfort.

These services influence health beyond the purely medical dimension. However, there are limits in our current system, especially for those with very severe conditions. If a program like PACE could more comprehensively include those individuals, it would play a meaningful role.

At the same time, caring for severely ill seniors is extremely demanding and requires high levels of expertise and emotional resilience. Adequate compensation and support for staff are essential for sustainability.

Because government funding is involved, some may perceive the system as business-oriented. While financial structures exist, I believe the core value must remain centered on genuinely helping vulnerable seniors. Especially for those who lack sufficient family support or are socially isolated, such services are essential.

Ultimately, these programs go beyond welfare provision; they support human dignity and the meaning of life in later years.

Thank you.

Professional 2. Senior Housing Coordinator
[Portion omitted]

Have you ever heard of the PACE program? If so, could you share how you first came across it?

Yes, I have heard of it once at a conference. I believe it was a conference for service coordinators. They introduced the program there. I became interested and looked into it, but I found that our apartment complex is not located within the zipcode served by the program, so our residents are not eligible. I remember feeling that it would have been beneficial if it were available to us.

It seems that the organization is making efforts to promote and introduce the program. However, I did not feel that it had deeply penetrated the broader community. I have heard that they are active in areas such as scam prevention, but I would not describe the outreach as highly visible. Some individuals with Medi-Cal appear to be enrolled in programs that operate in a manner similar to PACE. I understand that those programs provide support related to dementia and mental health as well. However, staffing appears limited, so rather than broadly covering everyone, they seem to focus on reaching out to those who are most in need. I am not entirely certain about the specific differences or operational details.

If this area were included in the PACE service region, what would you see as the greatest advantage?

For residents who have children, their children would likely manage the procedural aspects. Personally, however, I have often felt that such a structured system is needed. At one point, I even considered establishing a nonprofit organization myself and sought advice, but legal barriers made it difficult to pursue independently. I was told that it would require collaboration with stakeholders.

These days, more individuals remain unmarried, and issues such as return migration and living alone are increasing. I have thought a great deal about how to connect these individuals to formal

systems of support. After discussions with my supervisor, we concluded that the greatest barrier is language. Even when information exists, many cannot access it due to English limitations. If you have additional knowledge about this, I would also like to learn more.

If a PACE program were located nearby, how do you think residents would respond?

Currently, participants must receive an order from their primary care physician in order to attend a center. If PACE had physicians on site and provided transportation, individuals with more serious health conditions might choose that option. Many of our current center participants are relatively stable. Those who require more medical oversight might naturally gravitate toward PACE.

At the center, seniors often say, "I'm going to school," because they attend almost daily. It feels like part of their routine life. However, if there were a structure with on-site physicians, some might prefer it for the added sense of medical security.

Do you anticipate any tension between existing centers and a PACE program?

Centers have their own roles, and PACE seems more integrated with IHSS-type services. If PACE were introduced, residents might expect services such as meal delivery or in-home support. That could be a significant advantage. The challenge, however, is that many people still do not fully understand what PACE offers.

Were there any informational sessions or separate outreach events about PACE?

No, there were none. I only encountered it at the conference. Since our region is not covered, we were not invited to any related training. I doubt that Korean-language outreach has been extensive. Even among social workers, if the area is not covered by PACE, they may simply not know much about it.

Although the PACE program has existed for some time, it does not appear widely recognized within our community.

This may be a sensitive observation, but I sometimes feel that it may have been implemented with certain populations in mind. Officially, coverage is based on zipcode, but I wonder whether additional criteria exist. I am not certain.

Some seniors express frustration with physicians who they feel do not fully listen to them or who take a generalized approach without considering individual circumstances. Others, particularly younger physicians, are described as attentive and willing to make referrals when needed. In those cases, seniors may switch providers. However, many remain with their existing primary care physician because of familiarity and comfort. Changing physicians is not necessarily difficult procedurally, but emotionally it can be significant.

Some seniors say they lack information about physicians' specialties or geriatric expertise. It is not only about language; they also want to know whether a physician has sufficient experience with older adults.

Often, individuals simply follow the physician's referral. Even with interpretation support, the process can feel overwhelming. Social relationships also influence choices. Some choose centers because friends attend. There is a degree of group loyalty and even territoriality. In nearby areas, three centers operate, and participants rarely transfer. Relationships and seating arrangements are already established. New participants may feel excluded.

Do you think older adults may find it difficult to adapt to something new?

Yes. Even if a new program is established, whether individuals actually choose it is another matter. For those who have attended a center for many years, particularly individuals with dementia, transitioning to a new environment may be very challenging. Even if a program is objectively better, adaptation itself can be burdensome. Many now prioritize familiarity and stability over novelty or enhanced services.

[Portion omitted]

I personally feel that I need more supervision, especially in quickly identifying and connecting appropriate resources. In practice, situations require immediate response, and having organized, systematized information would be very helpful.

Language remains the greatest barrier. There are not many materials readily available in Korean. Even when I locate English-language resources and share them, I cannot complete the entire process on behalf of the resident. Ultimately, they must make calls or attend appointments themselves, and that is often difficult. I frequently feel the limits of what I can do as an intermediary.

Finally, is there any additional advice or message you would like to share?

I hope that any program could be tailored more closely to individual preferences. Some enjoy dancing, others prefer card games. While it is impossible to meet every need, providing some flexibility so individuals feel comfortable would be ideal.

Language remains the largest barrier. Programs may operate similarly overall, but seniors seek individuality and personal meaning within them.

Regarding dementia screening, I believe it would be beneficial if assessments were conducted more systematically and if general benchmarks or explanations were shared. While confidentiality must be protected, frontline staff need enough information to understand a resident's cognitive status and adjust approaches accordingly. Many seniors also wish to understand their own cognitive condition. Greater transparency could improve the quality of care.

Thank you.

Professional 3. Service Provider

[Portion omitted]

Have you heard of the PACE program?

PACE? I am not sure what that is. I have not heard of it before.

From my perspective, if Korean older adults have the will to use the medical system, they generally do not face major barriers. In the United States, some older adults live with their children, but I would estimate that 70 to 80 percent live in senior apartments. In those settings, there are social workers, sometimes attorneys, and even if they are not permanently on site, visiting social workers come regularly. Assistance is available. If individuals are willing, they can access services.

Older adults in this area receive services through senior apartment offices, social workers, religious institutions, and senior centers. Community organizations and churches provide volunteers. If someone is open and not socially withdrawn, there are many points of contact. The apartment manager, social worker, church, and volunteers all serve as resources. If someone visits a senior center, they can receive support. One must not be passive. It ultimately depends on personal initiative.

Korean older adults here are generally active. Information is not necessarily lacking. For example, even assistance with jury duty paperwork is provided if needed. If individuals register, attend meetings, and remain engaged, services are accessible. However, if someone says they cannot receive certain benefits without making an effort, that is a different matter. Of course, those who are physically unable to leave home are in a different situation.

Budget priorities are another issue. In Seoul, when the city reduces funding, children, individuals with disabilities, and older adults are often affected first. Los Angeles is similar. For example, meal delivery budgets were reduced. While congregate meals continue at centers, home-delivered meals for homebound older adults were cut. Those who attend centers can at least go to the market, but homebound individuals cannot. The decisions were made, and those affected often do not voice complaints. They are vulnerable and unheard. That is what I consider a blind spot. The media does not pay attention. These individuals, older adults living alone and unable to move easily, struggle daily to secure meals.

In my view, the issue is not necessarily a lack of institutional connections. Senior centers regularly exchange information. Over ten years of working here, I have observed that an 85-year-old who wakes early, attends church for breakfast, then goes to the senior center does not feel service discontinuity. The issue arises when individuals choose not to engage. Some may have personality differences or prefer isolation. Ultimately, it returns to personal choice.

If individuals seek services, they can find them. Many people are passive. Korean-language support is certainly limited, and cultural differences are substantial. In Korea, expectations often center on family responsibility—parents when young, spouses in adulthood, children in old age. Many expect daughters-in-law or sons to provide care. That cultural expectation is not inherently wrong, but in the United States, the norm is self-reliance. At age 65, many move to senior housing or manage independently. Cultural gaps are difficult to overcome. Some individuals expect to be served, which does not align with American norms. Adjusting to that gap is challenging.

For example, in Korea, family members might call a taxi for you. Here, they provide the phone number and expect you to call yourself. That difference requires adaptation. The responsibility falls on the individual. That adjustment can be difficult.

Language is a clear barrier. Older adults must learn to use smartphones or seek assistance. But how can some older adults do that easily? They must come to centers and learn. Korean-language support is insufficient.

I personally experienced this. I was transported to the hospital by 911. The physician spoke only English. I requested an interpreter. The hospital provided an interpretation device. Larger hospitals appear equipped to support minority languages. However, not every lower-level system may have interpreters. It seems that efforts are made at higher levels, especially in California.

After looking up PACE online, I think it sounds very good. However, Koreatown has many Korean markets, and large American markets struggle to enter. Adult day health centers operate as businesses. They receive reimbursement per participant. Approximately 150 participants may represent a break-even point. If a center loses participants, the financial impact is significant. Therefore, it is possible that some operators might resist the introduction of a PACE program. There are already many ADHCs competing with one another. If reimbursement is around $70 per participant, losing one person represents a tangible loss. I speculate that business interests could play a role in limiting PACE expansion.

This resembles a distinctly Korean-style environment, where cultural exchange with the broader system is limited. First-generation immigrants' perceptions create gaps. There may even be a mindset that limiting PACE access benefits existing centers. Information does not always flow easily. At times, community-level systems unintentionally block information. I believe information about PACE may have been restricted in this way.

Educational seminars and public sessions are needed. It is our responsibility to inform the community. Because PACE is government-based, people need to understand how it differs from private business models. Continuous effort to disseminate information is necessary.

Thank you.

Professional 4. Healthcare Provider (Nurse)

[Portion omitted]

Have you heard of the PACE program?

I did not know what PACE was. When I first heard about it, I could not really imagine what kind of care it provided. I heard that it was "all-inclusive," but I did not have a clear idea of how that care was actually delivered. Even with the name, I did not fully understand how everything was integrated.

When I explain it to older adults, they usually think of it simply as a day care center. They do not seem to understand the differences clearly. Because ADHC also provides exercise and activities, they assume it is similar, perhaps with a primary care doctor included. Even after explanations, many still understand it only at the level of a day care center. Some think about the convenience of having a doctor or dentist, and a few even misunderstand and assume that a physician is available 24 hours a day.

I feel that rather than through explanation alone, people would understand the program better by actually experiencing it. It is difficult to convey the full scope through description alone, especially since PACE often serves individuals with complicated conditions. Those with cognitive impairment may also find it difficult to fully understand what the program entails.

Are there any particular challenges or difficulties?

The biggest challenge right now involves family caregivers. Family caregivers often receive payment for providing care. If a participant enrolls in PACE, that payment arrangement may stop, which causes hesitation or even resistance within the family. We learned this during our preparation process.

In some cases, even when the caregiver is not a family member, participants may have a very strong attachment to that individual and say, "I cannot do this without my caregiver." Even when we explain that PACE provides transportation and home support, they first think about what they would lose. If there is an issue related to caregivers, many decide not to enroll.

Those using IHSS are particularly affected. If they enroll in PACE, they may no longer be able to use their IHSS caregiver in the same way, and PACE may not be able to match the exact number of IHSS hours they receive. For some, even if they believe the program is good, the caregiver issue becomes a barrier. In some families, adult children who are registered as IHSS caregivers preemptively discourage enrollment because they would lose income. Cultural factors may also play a role, as decision-making authority often shifts to adult children as parents age. It is a very sensitive issue.

For older adults without caregiver-related concerns, enrollment tends to be smoother. But when caregiver issues exist, recruitment becomes extremely difficult.

There is also the matter of changing primary care physicians. Some older adults hesitate to switch doctors, especially if they feel a strong personal connection. Many Korean older adults prefer to remain with a physician they feel comfortable with, even if insurance changes. The compatibility between the participant and the physician within PACE is therefore important.

Do older adults have particular preferences regarding physicians?

It varies, but many prefer physicians who are accommodating and not overly strict. If they request something, they appreciate it being provided without much resistance. Some physicians focus strongly on lifestyle counseling, exercise, and diet. While that approach can significantly improve health outcomes, some older adults find it burdensome.

Some prefer a simpler approach, such as receiving medication when they request it. For example, if they cannot sleep, they may ask directly for sleeping pills. There are certainly individuals who follow detailed guidance and benefit greatly from exercise and structured care, but others may feel that at their age, physical discomfort is inevitable and lifestyle changes are unnecessary.

Within Korean communities, word-of-mouth is powerful. At day care centers or churches, older adults share information about physicians and insurance companies. If they hear that a certain doctor provides specific services, they may change doctors. Similarly, if an insurance plan offers certain benefits, they may switch plans. I am not sure whether this is unique to Korean culture, but it seems particularly influential in this community.

What challenges do you anticipate for the PACE program?

We have explained that PACE offers comprehensive services. Participants will therefore arrive with high expectations. The question is whether we can consistently meet those expectations. Even if we assemble excellent providers and specialists, if a participant feels dissatisfied or if services are not delivered smoothly, there may be disappointment.

Because PACE involves interdisciplinary teamwork rather than individual outpatient care, coordination becomes essential. It is both a strength and a concern. We are not managing patients one-on-one; rather, we must integrate perspectives from multiple disciplines under the leadership of a primary care physician. That collaborative model may present challenges in communication and execution.

How does PACE differ from ADHC in terms of eligibility?

PACE requires that participants be able to live independently at home. Those requiring full nursing home–level care are not eligible. The program focuses on individuals with complex medical needs who nevertheless maintain functional independence.

For example, individuals in the early stages of dementia who are reluctant to participate in outside activities may still qualify. In fact, closer monitoring at a PACE center may be beneficial. However, once dementia progresses to a point where eating or basic functioning is severely impaired, nursing home care becomes more appropriate.

Some relatively healthy participants may feel uncomfortable being in the same space as individuals with more significant impairments. When older adults say they do not want to attend day care because “everyone there is old,” it reflects a psychological dimension. Those who are

mentally secure may not object, but those who feel vulnerable may resist. In such cases, scheduling adjustments or counseling may help.

What do you think about offering PACE services in Korean?

I believe it is an excellent idea. I have heard that other communities already have language-specific PACE programs, so it may feel somewhat overdue for the Korean community. While many participants in Koreatown have Korean primary care physicians, specialist appointments often require English. That language barrier creates discomfort.

If PACE can provide services in Korean and staff understand Korean culture, it becomes a significant advantage. It may not be necessary for 100 percent of staff to be Korean, but having a substantial proportion who can communicate in Korean would increase comfort and trust.

Is it difficult to recruit bilingual staff?

I am not directly involved in recruitment, so I cannot speak in detail. However, finding bilingual staff who are also highly skilled in their specialty can be challenging. For example, in physical therapy, it is important not only to speak Korean but also to be genuinely invested in patient improvement.

Some patients complain that physical therapists move quickly between patients without sufficient engagement. When therapists are sincerely committed to patient progress, patients can sense that. It would be ideal to recruit providers who genuinely care about helping patients improve.

Could there be tension between existing ADHC centers and PACE?

I have not personally viewed it as competition. ADHC provides programs and activities but does not include physicians. PACE integrates medical care and activities in a more comprehensive way.

Are there areas that should be improved during preparation?

The program is unfamiliar not only to patients but also to staff. More structured education about PACE would be helpful so that we can clearly explain how it differs from existing models.

During preparation, we have realized that issues related to caregivers, insurance, and low-income participants require further research and strategic planning. Financial incentives associated with insurance plans strongly influence decision-making. Couples often move together, which amplifies the financial impact. Given the tight-knit nature of the Korean community, information spreads rapidly and group decisions often follow. Anticipating these patterns would help in recruitment and outreach strategies.

How can awareness of the program be increased?

We explain how PACE differs from a typical day care center but try to avoid creating resistance. Outreach through churches, senior apartments, and word-of-mouth from current participants may be effective. Once participants understand the program, they can share information within their networks.

What are the basic enrollment criteria?

Participants must be 55 years or older, have Medi-Cal, and require a nursing home–level of care while still being able to live independently at home. Individuals undergoing active cancer treatment or requiring hospitalization are not eligible. Those with severe cognitive impairment who cannot follow up reliably may also be difficult to enroll. Dementia eligibility depends on stage.

Is there stigma around nursing home placement among Korean families?

Yes, there is significant stigma. Often, families attempt to manage care at home until it becomes too difficult.

Do you have any message for future PACE providers?

Although we have not yet fully launched, I hope that through this program, patients will receive substantial support and experience improved health. It will require significant effort from staff. The caregiver issue remains particularly complex. While financial considerations cannot be ignored, from the patient's perspective, the program has strong potential.

At the same time, providing such comprehensive care to one individual is not simple. It differs greatly from outpatient care and will likely involve unforeseen challenges.

We are still learning. The fact that we are uncertain reflects how new PACE is within this community. I sincerely hope it succeeds.

Thank you.

Professional 5. Social Worker

[Portion omitted]

There are many ADHCs, right? Have you heard of PACE?

Yes, I have seen job postings for PACE. Even in ADHC settings, licensed staff are needed, but it seems that PACE requires those licensed professionals to be on site more consistently. Since some participants have not only Medi-Cal but also Medicare, it seems like they have expanded staffing expectations. With the older adult population increasing, it also seems that more PACE sites are opening.

Have you ever received clear, formal information about PACE?

Not really. My work is not exclusively focused on older adults, so I have not received a clear briefing. But I was interested, and as I looked into it on my own, I learned more about it.

PACE is interdisciplinary, but ADHC is already well established. How do you see that relationship?

If we focus on Korean PACE, ADHC largely serves people with Medi-Cal. PACE, on the other hand, includes older adults who are 65 and older and therefore have Medicare, including those with both Medicare and Medi-Cal. That makes the potential market much broader. Some older adults also have difficulty obtaining Medi-Cal, so in that sense PACE seems designed to be available to older adults more generally.

If we think specifically about Korean older adults, there are also financially stable seniors. Those individuals could potentially be absorbed into PACE, especially if higher-quality services are provided. However, if the level of services feels similar to ADHC, people may ask, “Why would I switch, especially if it requires changing my doctor?” Those who have more resources also have alternative options, so PACE would likely need to offer clearly differentiated, higher-quality services to be competitive.

At the same time, I do think many Korean older adults are on Medi-Cal, which may be part of why ADHC has been functioning well. Many older immigrants did not have long employment histories here. Some came later in life to help with grandchildren or through family sponsorship, so it may have been difficult to maintain long-term employment. Some have Social Security through small businesses, but many are covered through Medi-Cal either through their adult children’s support or their own enrollment.

So expanding options for those with Medicare is valuable, and it is also beneficial that those with Medi-Cal would have PACE as an additional option.

Within the Korean community, how would you describe the level of understanding about PACE?

When I mention PACE in my own setting, some people say they have heard of it. It does not seem completely unfamiliar. Many of them live in senior apartments, and once information spreads, people seem to become aware. However, most appear to know it informally rather than understanding eligibility requirements clearly.

Some people also say they cannot attend ADHC because they do not have Medi-Cal. If they hear that both Medi-Cal and Medicare are accepted, they may view it positively. They usually ask practical questions, such as whether they would need to pay or whether they qualify, because that is important.

Do you currently provide counseling to Korean older adults?

In the past, when I visited nursing homes directly, it seemed that Korean older adults tended to go to certain specific facilities. There are many Korean older adults in Koreatown. I do provide

counseling to Korean clients, and among those aged roughly 60 to 75, about half are Korean. Many seek counseling for mental health concerns.

Some of these concerns relate to adult children. For example, the children are now grown but struggle with employment. Many families came through investment visas or to create educational opportunities for their children, but outcomes are not always as expected. In some cases, immigration status remains unstable: the parent may not receive permanent residency, or the child may have DACA while the parent's status becomes precarious.

Some clients remain responsible as the primary provider even into their 70s, sometimes working cash jobs. The burden can be heavy. Immigration-related stress is significant. At the same time, physical frailty increases, and after age 65, dental issues and other health needs become costly. Medi-Cal may cover preventive care, but dentures or implants often require out-of-pocket payment. Without stable legal status, even access to benefits becomes difficult.

Chronic pain, injuries such as from traffic accidents, and declining health can contribute to persistent depression. Family conflict is also common. Some have little support from children. Some struggle with meals and rely on one meal per day. For individuals on dialysis, dietary management is required, but many say it is difficult to follow.

In Koreatown there are many Korean-language services. How are people using them?

Compared to other states or regions, the availability of Korean-language services in Koreatown is a major advantage. For example, a dialysis patient I worked with was trying to apply for disability income. When they contacted social services, they were able to receive interpretation support over the phone from a Korean-speaking staff member, and they found that very helpful. In Koreatown especially, services seem to function relatively well because the Korean population is large.

Many people even move here from other states because Korean-language hospitals, services, nurses, and social support are limited elsewhere. Even if quality services exist in other regions, language barriers often lead people to relocate. Some also move from other parts of California to Koreatown.

For example, in Orange County, adult children often need to drive parents to appointments. In Los Angeles, the infrastructure is better, so older adults can attend treatment independently. That independence also connects to dignity. In areas where everything is spread out, people may end up staying at home, which can feel isolating and restrictive.

So Los Angeles may be better for independent living?

Yes. Even if it looks physically difficult to us, many older adults see walking, pulling carts, and moving around on their own as part of living independently. Some also have friends nearby. For those in senior apartments, there seems to be substantial support.

ADHC is also multidisciplinary, and a physician may be listed, but in many cases physicians do not come regularly. Staffing is structured around required positions, whereas my understanding is that in PACE, physicians are required to be on site. That feels like a more medicalized model, perhaps somewhere between ADHC and nursing home care. It seems designed as a response to the growing older adult population. Since it is not restricted only to Medi-Cal participants, it may benefit a broader group of older adults.

Korean older adults often value having direct access to a physician. Since it is not always easy to see a doctor, the presence of a physician could be a major advantage.

If you were to work as staff within PACE, what do you anticipate?

Social workers often become part of interdisciplinary teams, but in medical settings, the environment can be heavily medicalized. Social work can feel vaguely defined. Sometimes people assume that anything non-medical automatically falls to social work. In that context, clarifying the psychosocial scope and contribution becomes part of the social worker's role.

Medical staff may focus on clinical indicators, whereas social workers often identify the reasons a patient cannot attend or adhere to care, such as lack of family support, lack of transportation, or depression. Social workers often advocate for patients in team meetings. For example, if medication adherence is poor, the social worker may identify contributing factors such as cognitive impairment or lack of support and help coordinate resources. Since social work is closely tied to case management, teamwork is essential.

For PACE to become established in the Korean community, what needs to improve?

First, eligibility criteria must be clearly communicated so that anyone can understand who qualifies. Second, it needs to be explicit how PACE differs from existing services. If diverse and specialized services are visibly available, that would help.

A major question for many patients is whether they would need to change their primary care physician to enroll in PACE. That concern does not exist with ADHC. Because there is overlap in services such as transportation, blood pressure checks, and diabetes monitoring, it is important to clarify what is duplicated and what is truly different.

In ADHC, attendance frequency is also tied to severity: more severe cases may qualify for five days, whereas others may receive approval for only two days. When I worked in ADHC, I often observed a business mindset, such as encouraging someone approved for two days to attend five. If gaps exist in what ADHC can offer, it would be ideal if PACE could complement rather than simply compete.

If mental health services are lacking elsewhere, PACE could fill that gap. If transportation is provided but someone prefers to drive, parking availability may matter. Ultimately, higher-quality medical care and physicians would encourage voluntary, active participation. If services appear similar to ADHC, it may look like simply taking participants away from existing centers.

Word-of-mouth through Korean churches and other community hubs may be effective, since many older adults socialize in consistent locations. There are also physicians who accept only Medi-Cal. If there are Korean-speaking coordinators within those groups, outreach to them might be useful. Physician-to-physician outreach could be difficult, but connecting with coordinators may be more feasible.

Thank you.

Professional 6. Service Provider

[Portion omitted]

Have you heard of the PACE program?

Yes, I have heard of it. Once it is actually operating, I would like to observe it more closely and understand how important it could be and how much it could help older adults. At this point, I do not feel that I know enough about it yet.

PACE is a government-supported model intended to help older adults remain in the community without entering a nursing home. From your perspective in the field, if a program like this were introduced, what aspects seem promising, and what concerns might you have?

If such a program enters the Korean community, I think it could be especially beneficial for older adults who have difficulty communicating and who need support. It would be helpful if they could get where they need to go by taking a taxi or walking, without the burden of having to rely on someone or call their children every time.

It would also be meaningful if the program could become a space where older adults can comfortably share what they need in daily life, what they are lacking, and what feels difficult, and receive support in an ongoing way. Ultimately, I think what matters is having the right people, the right professional expertise, and real experience that allows staff to accurately understand what each older adult truly needs. If those elements are in place, then older adults can be guided in the right direction.

Being able to receive services in Korean is clearly important, but it must be paired with high-quality medical care. It should not be enough that communication is easy. Older adults need accurate diagnoses, appropriate treatment and recommendations, and enough information to understand their conditions and care plans.

If that information is not provided properly, people can lose years. For instance, if someone receives an incorrect diagnosis for pain, they may accept that explanation and spend time without receiving the care they actually need. For older adults, time is extremely important. They need to identify what is happening as early as possible and receive timely care. Missing that window due to inadequate evaluation or treatment would be harmful.

If older adults with diverse backgrounds and health conditions participate together, do you have any concerns about the relationships, group climate, or interpersonal dynamics that may emerge?

[Portion omitted]

Many people come here because they enjoy meeting friends and connecting socially, even though they may initially come for the program itself. PACE may also offer activities, but I think our center functions as something like a second university for older adults. It is a place to start things they wanted to do earlier in life but could not due to time or circumstances, such as learning to paint or taking singing lessons. Many seem to experience this as a place of learning.

In contrast, I imagine PACE may feel somewhat lighter than our center, in the sense that it may focus more on helping older adults spend time in a structured way, move their bodies, and remain active, which can itself support health.

If participants include individuals who need more intensive medical support, do you have concerns about the overall atmosphere or the ways healthier participants might respond to that environment?

Yes. People would not be sleeping there; they would return home at the end of the day. So their life would still remain centered in their community and home. However, if there are many people who are very ill, the atmosphere could become heavy, almost like a hospital environment. Even individuals who are not severely ill can sometimes feel "sicker" when they are surrounded by that kind of environment. I hope that does not happen.

Instead, I would hope to see people gradually improve. If ten people come in and most of them begin to move better, function better, and live more comfortably over time, that would be a very meaningful outcome. In that sense, PACE could become truly significant for those who previously could not use existing centers. Some might feel, "This program gave me my life back," and experience real improvements in quality of life. I hope many people will have those positive experiences.

How do you think about food and meal provision, including preferences and feasibility?

We offered meals for a period of time. Initially, it was not Korean food. For about two months, we offered Korean meals, but due to budget constraints we returned to a standard menu. To be honest, many people did not find it appealing. Over time, some said it tasted bad or felt like hospital food. Preference seemed to decline.

Many older adults who come to our center are quite active, relatively healthy, and cognitively clear. They do not come for the food. In fact, people living nearby were more likely to pick up meals. Those who use the center typically attend their programs, then leave for other activities and return later.

In PACE, however, food would likely be much more important. Diet management tailored to a person's health condition would be necessary. It is not simply about providing meals, but about supporting structured dietary plans for each individual. In that sense, it seems closer to a model that manages daily living comprehensively before care needs escalate to hospitalization.

And yes, for conditions such as diabetes, nutrition and avoidance of certain foods matter. Beyond preferences for Korean versus American food, attention to nutrients and restrictions would be essential. Older adults also need meals prepared for them. When it depends on their own effort every day, it becomes burdensome and adherence declines, even if they know what they should eat for their health. PACE may need to provide ongoing guidance and practical support, including what to eat at home, and possibly food preparation support that helps people follow through.

Although PACE provides health and medical services, the social meaning of relationships and community also seems important. For older adults who live alone or only with a spouse, what is your perspective on the social function of such a community-based program?

It would be very beneficial. Many older adults stay home because of illness. Even when they want to go out and do something, they may not have the conditions to do so. If PACE helps them understand their health status, clarifies what they need, provides structured care, and offers natural opportunities to interact with others, that could be deeply meaningful.

I have seen cases where someone was living quite healthily, but after a fall and injury to the leg or back, they became mostly homebound. Over time they may regain some mobility but remain uncomfortable and limited. If that person could receive physical therapy through PACE while also learning to paint or participating in other activities, it would be extremely helpful. It would allow them to feel vitality and meaning during recovery, not only clinical treatment.

I would also hope the environment is bright, comfortable, and welcoming. If there is an outdoor garden view, plants, or flowers, that would be ideal. Many older adults enjoy caring for plants. In my experience, when such spaces exist, older adults participate voluntarily and find enjoyment in actively tending them.

[Portion omitted]

Some people have raised concerns about the name "PACE." What do you think about that?

I am not sure. From the perspective of Korean older adults, the name needs to be immediately understandable and memorable. If the name feels confusing or difficult to remember, people may simply move on and not consider it. I think the name itself matters.

As you mentioned, Korean-language communication and comprehensive care in one place are major strengths. At the same time, in tight-knit communities there may also be conflicts

or tensions among participants, and even within interdisciplinary teams. Based on your experience, what advice would you offer for planning and implementation?

In any organization, conflict can arise at multiple levels: leadership, staff, participants, and between participants and management. Ultimately, I think leadership is the most important factor. If leaders delegate roles and responsibilities clearly, and if they communicate well by staying connected to what is happening on the ground, many problems can be reduced. It may sound obvious, but strong delegation and genuine effort to understand the field can help the whole organization function better.

Each person needs to maintain professional boundaries and expertise while doing their best. Whether someone is in a senior role, middle management, or frontline work, everyone needs to continually reflect on why they are there. Ultimately, the people being cared for are older adults and patients. If staff work with the mindset that these are their own family members, the work can become emotionally easier and feel more genuine.

From my experience running an organization, I emphasized that point when speaking with staff. If the team understands what each person truly needs and provides clear direction and guidance accordingly, the organization tends to run better.

[Portion omitted]

In areas with many Korean older adults, it sometimes seems possible to live relatively independently even without Korean-speaking children nearby. In that context, how do you view changes in family roles and adult children's involvement?

Some adult children say their mother looks much happier after attending the center. They say her expression becomes brighter and she seems more harmonious through social engagement. Some say, "Now my mother has more friends and will feel less lonely," and they seem reassured.

Especially among adult children in similar age groups, many view their parent's attendance very positively. In that sense, these spaces are not only for older adults but also provide reassurance for adult children. When centers host events and share meals, or provide small gifts and necessities, adult children often feel relieved. When children live far away, in another state or another country, simply knowing that such a community exists seems to offer significant comfort.

A community can create an environment where older adults are able to live independently without relying entirely on their children. Of course, when health declines, adult children may need to re-engage, but that is not always easy. They have their own lives, and physical distance can make involvement impossible. PACE may help by providing medical care and home visits, which can also reassure adult children.

Given the range of services PACE aims to provide, would staffing be a major issue?

[Portion omitted]

Overall, staffing seems to operate based on ratios linked to the number of participants. From what I have learned, eligibility is limited to those living within certain geographic boundaries, and participants generally need to be enrolled in both Medicare and Medicaid and meet specific levels of medical need. If those conditions are met, it seems that a physician must confirm and approve enrollment.

Another important point is that enrolling in PACE may require changing one's primary care physician.

That raises the possibility that some primary care physicians might hesitate to encourage transition to another program, because they want to retain long-term patients. From the patient's perspective, switching providers after years of care could also feel burdensome, and some may hesitate because they want to maintain familiar relationships.

[Portion omitted]

I think it is better to identify both strengths and concerns in advance, and to approach implementation by offering solutions proactively.

From what I see, I feel that readiness is already quite strong. I see people who appear to have difficulty walking still come to the center. When meals are provided, someone may arrive early and wait. I once told someone with a walker, "If this is difficult, you can apply for meal delivery," and the person said, "Coming here to receive food is my joy." They waited for over an hour and said that simply walking to the center and returning home is meaningful.

When I think about people who remain at home, I see more clearly why the role of a center is important. If it is a day off and there are no special plans, anyone can end up staying at home. Older adults are the same. After retirement, without specific commitments, there may be no reason to go out. So one of the most important roles of such spaces is that they bring older adults out of their homes.

It is not only about how long they stay. The process itself matters: leaving home, coming to the center by walking, taxi, or bus, meeting friends, smiling, eating together, talking, and then returning home. That cycle provides an important daily structure.

I also think many people are invisible. They are not seen because they are sick and remain at home, so it can feel as though they do not exist, but that is not true. There are more people like that than we realize. That is why I think this type of program is already needed, and needed urgently. If transportation is provided, many people would participate without worrying, "How would I get there?"

If PACE begins to serve older adults who cannot attend existing centers due to mobility limitations, are there safety concerns that should be anticipated?

Yes. I think there may need to be safety regulations similar to school zones, such as speed limits and safety rules. Even inside the building, stairs and walking paths would require careful planning.

I have heard of an incident where an emergency occurred during a time when no management staff were on site. That underscores how important safety monitoring and rapid response systems are. The current center population is relatively younger, but if a more medically vulnerable group were being served with more professional care, those emergencies might be handled differently. That is why the need for a system like PACE, with ongoing medical observation and support, feels even more important.

[Portion omitted]

I think it is ultimately similar to a business situation. If I run a restaurant and a new restaurant opens nearby, I may worry about losing customers. That dynamic can exist in healthcare as well. However, the most important criterion should be what the patient needs. Even if someone is "my patient," if another system is more appropriate, we should be able to recommend that option.

For that to happen, providers need to understand how the program operates and what care it actually provides. With that understanding, clinicians can decide, "This would be the best support for this patient," and recommend it. Even if that means they no longer see the patient directly, professional ethics require guiding patients toward the most appropriate option.

Day care operators could continue their existing programs while also operating PACE, or collaborating with other organizations. If they determine that a participant would be better served through PACE, they could refer that person naturally.

If you could offer advice to someone who is planning to operate PACE, what would you emphasize as the most important preparation?

Ultimately, I think building a strong team is the most important factor. Staff need professional expertise and the ability to apply it conscientiously. At the same time, they need genuine respect for older adults and the capacity to provide compassionate care.

In my experience, I have seen providers who are highly skilled but emotionally cold in the way they treat patients. Technical competence matters, but there is a clear difference between care delivered with empathy and careful explanation versus care that feels detached. For a program like PACE, it is not enough to recruit only competent professionals. It is essential to build a team that combines expertise with empathy.

In that kind of environment, families may say, "My mother became much brighter after joining PACE," or "My father became healthier and his expression changed." If older adults themselves seem happier and their outlook becomes more positive, that is what successful operation looks like.

If the program is truly helpful, extensive advertising may not even be necessary. When participants say, “I went there and it was really good,” that message spreads naturally and becomes the most credible promotion. That is why I hope the program can provide sustained, consistent care. To do that, it requires selecting the right people and creating conditions that allow them to stay and work together over time.

In the end, the hardest part is often people. Leadership is difficult largely because of people. More than the work itself, relationships within the team often become the greatest challenge.

Thank you.

Professional 7. Social Worker

[Portion omitted]

When you look at the PACE program, it can include not only individuals who have both Medicare and Medicaid, but also people from diverse backgrounds. That seems like it could increase accessibility, and it also seems like a strength that multiple services are provided in one place. In the field, to what extent do people know about this, and how well is it actually understood?

To be honest, we have been exposed to PACE little by little through training materials for several years. Each year, when Medi-Cal policies change, we receive related trainings, and PACE was included. But it did not feel concrete. I had never seen it directly, and I did not know how it operates in practice. So in the training materials, it was not treated as a major focus. Often it was mentioned briefly as something like a “community-based service.”

The issue is that “community-based services” includes so many programs. In that category, PACE was perceived as just one option among many. I think we understood it as one more program run through a managed care plan.

More recently, I had a chance to work with staff who work in PACE. Through that, I felt that I needed to confirm what the service actually is. I visited a PACE center, and only then did I realize that it is something we really need to know. Before that, it was something I read in materials and moved past. But after seeing how it actually operates, it felt like a highly important program.

After that, we started explaining it again internally, and we shared information with staff that this type of service exists. We also visited newly established centers and began to understand the structure more concretely. Later, I realized that PACE was already included in the standardized forms that Medi-Cal beneficiaries are required to complete. Internally, we even said, “If it is this important, shouldn’t there have been more systematic training specifically for practitioners?” At the same time, we also reflected on whether we had simply not recognized the earlier training sufficiently.

In particular, what felt confusing was that the distinction between PACE and other long-term services we already knew was not clearly explained. For example, how it differs structurally from CBAS, home health nursing, hospice, or IHSS, and what makes it distinct, did not come through clearly in the field. In practice, CBAS can look quite similar in terms of services. Both involve care planning, transportation support, and involvement of nurses and social work staff.

PACE emphasizes integrated services, but if we do not explain what is different and how it is different, people in the field will naturally think, "Doesn't CBAS already provide integrated services?" Ultimately, the core distinction is probably the interdisciplinary, team-based model, but because that was not emphasized sufficiently in trainings or materials, it felt difficult to differentiate services.

About how many years ago do you think you first encountered PACE through official notices or formal materials?

I think I first encountered it about four to five years ago. During that time, there were no clear directives or systematic guidance coming down from leadership. But in the training materials, the term "PACE" began appearing more frequently and more prominently. There were statements like "PACE exists" or "it is an all-inclusive service," but because we had never actually seen it in the community, it was hard to connect those words to something concrete.

If there had been a center available at that time, we could have said, "Oh, this is what they mean." But without having seen a real model, we had no reference point. I think social workers, community practitioners, and even hospital staff who handle referrals likely experienced something similar. If you have never seen or experienced a service, it is difficult to explain it to someone or connect them to it. I was only able to explain it more clearly after I visited and saw operations and services directly.

There was one case I remember. A person was using a long-term care center, but their spouse had dementia, so it was hard to leave the spouse alone at home. They arranged a caregiver temporarily so they could come to the center, but even while they were at the center, they said they felt uneasy the entire time. I told them it might help to exercise and meet people to relieve stress, but their main concern was different from what I initially assumed.

What they struggled with most was the wish to take responsibility and care for their spouse until the end, combined with anxiety about whether the current care arrangement was truly appropriate for their spouse. They were also afraid of having to make major decisions alone, repeatedly, in a situation where 24-hour help was needed. Family support was also limited. Listening to that, I thought, this is exactly the kind of situation where a service like PACE may be needed.

I tried to explain PACE, but for them it did not feel concrete: how to change the primary care provider, how services would actually be delivered. It seemed like they interpreted it as "maybe meals get delivered," and it did not lead to an actual linkage. Looking back, they had never seen the program, never toured it, and had not heard any peer stories of using it. That likely shaped how they received the information.

Also, the existing long-term care structure sometimes approves up to five days per week, but in practice some people use only three days, which can create operational burden and may not match actual needs perfectly. PACE includes care that continues beyond the center and extends into time at home. In contrast, some existing services stop at the end of center hours and do not cover life after that. In that sense, the strengths of PACE are clear, but because there are still few user experiences circulating, those strengths are not being shared or explained well in the field.

It sounds like low understanding of PACE among those who provide care is a major barrier.

Yes. PACE is fundamentally a service for people who need long-term care. But among that population, many have difficulty making healthcare or care decisions on their own, and families or caregivers end up making decisions. The problem is that families often do not have sufficient information either.

I heard about a case where a staff member who worked in PACE transferred to a PACE center in another region. They said that center is already operating at the scale of hundreds of participants. It seems that in that region, community awareness and outreach are relatively well established, and PACE coordinates medical appointments and schedules in a way that substantially reduces the family's burden.

Hearing that made me think again about how central trust and linkage with families are for PACE to function well. Families need enough information and communication to feel safe delegating decisions.

How do you think that region became so established? Could it relate to community characteristics, demographics, or existing networks?

It could. From what I heard, that region had a high proportion of a specific immigrant community, but there were almost no Korean participants using the service. I understand that many participants had Medi-Cal, and some did or did not have Medicare. Possibly, once early users accumulated, experiences and cases naturally expanded. It is also possible that the environment had fewer existing adult day health centers, which may have made PACE relatively more salient.

In contrast, in the Korean community, adult day health centers are already strongly established. So PACE may be perceived not as a new model, but as just another "center." If its distinctiveness is not communicated clearly, people who are already using services have little reason to switch.

PACE is not necessarily a structure where participants must attend a center five days a week, and it includes services that extend to time at home. So it seems very appropriate for people with higher levels of care needs. Yet in reality, some people in their 80s or 90s, even when their health is poor, end up in situations where they have to attend a center five days a week, and that creates difficulties. Adult day health centers also have limits in accepting individuals with significant functional constraints, such as those who cannot use the toilet independently or who

are too frail to participate in activities. Because staffing ratios are legally defined, when one individual needs intensive care, it can burden overall operations. As a result, some people discontinue center use and remain at home. When we see those situations, we sometimes say that those individuals might be more appropriately linked to PACE.

I also heard that in terms of eligibility, adult day services and PACE may not be radically different, in the sense that both serve people who require a certain level of help with daily living. I also heard that some people have transitioned from existing services to PACE. Still, the most frequently mentioned difference seems to be whether the person must change their primary care provider. I also heard that some models allow partial continuity with existing networks, depending on how they are structured. In some cases, PACE is introduced as part of insurance products, creating connections through insurance agents.

However, at this point, comparisons often focus on visible services such as meal provision, rather than structural strengths such as 24-hour integrated care or home visiting nursing. That contributes to a sense that the core value of PACE has not been communicated sufficiently.

In Koreatown, how well known do you think PACE is?

I think many people may not know the name at all, and "PACE" may feel unfamiliar. That is why I recently felt that when we do Medi-Cal and Medicare education at churches, we should include PACE more concretely and promote it together. Because PACE is already listed in Medi-Cal selection forms, when I pointed that out and explained it, quite a few people listened with interest.

What many people say is that they do not want to spend the end of life in a nursing home. They want to remain at home if possible. For them, the idea of receiving nursing-home-level services at home can feel like a major advantage. Some also see it positively because children can come and go and participate in care in a manageable way. After hearing the explanation, many people said, "This is good."

But the problem is that almost no one around them has actually used PACE. Without user experiences, trust is difficult to form. Many people have also had negative experiences with insurance or HMO systems, and they receive frequent education about scams, so they may be more cautious about unfamiliar services. Another problem is that even some professionals who would need to connect people to PACE do not fully understand it. If the providers who need to make referrals do not know the service well, linkage becomes difficult. I have heard that PACE is very active in other regions, but I have not confirmed that directly, so it remains secondhand information. That makes me think that seeing concrete cases and sharing lived experiences is essential.

Do you think language and cultural factors also shape why there are fewer operators or users in the Korean community?

Yes. I do not think it is accurate to say that there was no mainstream education at all or no information provided. But I do think there were problems in how the information was

communicated and translated. Because PACE was grouped under the broad label of "community-based services," its unique features and distinctions were not made visible. Within that category, because so many programs exist, PACE may have been diluted and perceived as simply one option among many, rather than as a distinct model.

Also, in Koreatown, existing adult day health centers are very strongly established, and it may not be easy for alternative nonprofit models or new senior-focused community institutions to compete. There are many centers with substantial enrollment, and many older adults are already using them stably. That can reduce the perceived need to seek a new model actively. In this structure, even when senior centers exist, they may not always be staffed with sufficient specialized professionals.

In addition, there is a trend where service coordination roles within housing settings have become stronger, and some social services are increasingly absorbed at the apartment level. That may contribute to a sense that community-level, senior-centered integrated institutions are being reduced.

I think it needs to be clarified more clearly that PACE is intended for individuals with higher levels of care need. Right now, it is often perceived as competing with adult day services, and comparisons focus on meals or eligibility similarities. But in my view, PACE is more appropriate for people who cannot easily use adult day services and who need ongoing medical and social support while remaining at home. If those individuals can receive care through PACE, it can help fill a gap that existing services do not reach well.

How could we reach those individuals more effectively?

If possible, it would be best for primary care providers to make referrals directly. But I am not sure how well primary care providers understand these services, or whether the structure supports referrals smoothly. That is why I think apartment social workers, service coordinators, and other community-based practitioners should also know about PACE. They often understand older adults' living situations most closely and can identify those who are too healthy for certain services and those who are too frail and need more support.

Whether they are apartment social workers or managers, if they can observe older adults' status and refer them at the right time, services could be connected more effectively to those who truly need them.

What would help activation and broader uptake?

In some regions, PACE may have become established because there were not many existing adult day services. In other regions, PACE may operate well even when multiple sites exist. In those contexts, PACE may function more like a clinic or hospital-based structure, so people enter through medical visits and then naturally use the attached services. That structure can make access easier.

For individuals with only Medi-Cal, choices can be limited, so receiving multiple medical services in one place may be especially appealing. When it is difficult to find a primary care provider, and when PT or OT is hard to arrange separately, having those services bundled can be very attractive.

I also think it may help to clarify differentiation from adult day services, not as a competition but as a staged linkage. For example, someone may use adult day services while they can attend, but as it becomes harder to come to the center, they could transition to PACE. If both services are framed as part of a broader medical continuum, moving to a more appropriate service as needs change could feel natural.

Of course, providers may not always agree, because there are different interests. But if PACE can also send someone back to adult day services when that is more appropriate, and if adult day services can refer someone to PACE when needed, a more participant-centered flow could develop.

Ultimately, the key is education on what PACE offers differently: that it is not necessarily five days of center attendance, that home-based meals and nursing can be provided, that hospice can be integrated when needed, and so on. I personally began looking into PACE more because I heard it more often. But I do not think all social workers do that. Many provide support mainly to patients who come to them. Adult day services sometimes mention PACE exists, but I am not sure how well the differences are understood, even when transitions are already happening.

I also think there is a perception that PACE is financially more advantageous. I have heard that many adult day operators wanted to run PACE, but that it was not easy due to conditions such as physician approval. Some attempted and did not proceed. In the field, there can be talk like, "PACE is more profitable," or "We should not lose participants to PACE." At the same time, adult day services themselves are financially strained: labor and operating costs have increased, but per-participant reimbursement has not increased proportionally, making it harder to break even.

Given this context, some people also believe that adult day services and PACE should be viewed not as opposites but as services that need to coexist. If both require medical approval, it becomes even more important to communicate clearly to medical providers which service fits which situation, and to link people accordingly.

So in the end, more education and communication are needed, including for physicians, on how to determine whether adult day services or PACE is more appropriate.

From a primary care physician's perspective, referring a patient to another program could feel like losing the patient.

Yes, physicians may have ambivalent feelings. I also heard that hospice referrals are often not made proactively for similar reasons: referral can feel like ending the relationship. It may be difficult for a physician to send away a patient they could otherwise continue seeing for years.

I also heard that in some regions, adult day services have become saturated and have expanded into other areas. In those areas, participants may be much more impaired, with significant cognitive or emotional difficulties, making center use challenging. That makes me wonder whether in some places adult day services have been covering even relatively high-functioning individuals quite broadly, including people who may not strictly need center-based services.

Right now, even opening one new center can create a very sensitive environment. If a center expands or uses aggressive marketing, many older adults may move. Adult day services can market relatively freely, whereas I heard that PACE has much stricter marketing regulations. Public advertising and direct outreach are limited, and information sharing may be largely constrained to provider-mediated channels. That seems to be another barrier to spread.

Personally, I wish there were more opportunities for families and communities to encounter the information and decide. If people who see older adults in everyday life could also participate in decision-making, linkages might be more appropriate.

Another thing I noticed is the importance of visibility. Facilities that are physically visible on the street naturally prompt curiosity: older adults pass by, wonder what the place is, and may stop and inquire. Sometimes they even wait through construction delays because they have become interested. But PACE is often not as visible, so older adults may have difficulty even recognizing that it exists.

These structural factors may be compounding, leaving PACE with low recognition and accessibility despite clear need.

At this moment, when PACE is trying to become more widely known, what should be improved in communication and information delivery between organizations?

I think nearly all of it needs improvement. I only learned recently that PACE was included in Medi-Cal forms, but it seems that even some PACE-side staff may not have been fully aware of that. Formally, self-referral is possible, but realistically, how likely is it that an older adult will select PACE on a form if there has not been sufficient education? That seems very unlikely.

That is why education centered in senior apartments would be very helpful. Apartments often include people with higher needs, and information can be delivered directly in the context of daily life.

I also hope PACE is framed not as competing with adult day services but as collaborating. Not a structure of taking people away, but a flow where each service links people according to role and fit.

Realistically, it does not seem that clear government guidance or systematic training will be provided from above. So for now, the most realistic pathway may be that those of us who learn about it introduce it one by one, and then older adults' lived experiences spread by word of mouth.

[Portion omitted]

One difference between older adults in Korea and the U.S. may be how they view late life and the end of life. How do you see that affecting how PACE should be explained?

I think one of the biggest differences is how people conceptualize aging and the end of life. In Korea, facility-based options such as long-term care hospitals, nursing hospitals, and senior towns are relatively common, and many people spend late life or die in those settings. In the U.S., many people remain in the community, living in senior apartments or at home with family.

For Korean older adults living in the U.S., the desire to remain at home until death may be stronger. The idea of dying in a hospital environment where language is not accessible may feel deeply undesirable, and that may be a very natural and strong preference. In Korea, there can also be cultural beliefs that dying at home lowers property value or is treated as an "incident." In contrast, in the U.S., dying at home can be viewed as a natural choice.

I think it is important to explain and contextualize these cultural differences. Many older adults seem to carry an image of dying alone in a facility, not seeing family often, lying in a cold bed, and not being able to communicate. When you actually see those environments, it is not hard to feel, "I would not want to die that way either."

At home, people may feel they have some ability to organize their life and close it in a way that reflects their choices and dignity. The possibility of receiving professional support while adapting the home environment to provide hospital-like care is also an important element. In that sense, PACE may be explained not simply as a service, but as an option for how one wants to live and die in later life. The message may need to be: "This is the kind of later life I want to choose."

If there is an opportunity to communicate PACE, I think it would be helpful to convey that value and direction. I have seen many people say they want to die at home, but I have almost never seen someone say they want to die in a hospital.

Are there concerns about staffing and operational management?

CBAS can be seen as operating within a broadly similar service framework. But because CBAS often lacks financial flexibility, PT and OT can be very limited, and sometimes activity coordinators partially compensate for those functions. In contrast, as I understand it, PACE involves direct intervention by PT and OT, and in some cases even home visits. If so, PACE could provide more individualized support aligned with each older adult's condition and needs.

Another reality is that in CBAS, PTs often have little opportunity to talk with older adults or build relationships. Because sessions are short and limited, there can be constraints in understanding the broader life context beyond physical function.

How feasible is hiring bilingual staff?

Realistically, it may not be easy. In CBAS, it is often difficult to create stable full-time positions, which makes it hard to reduce turnover and sustain high-quality services. For people to continue working with a sense of mission, the environment has to support that, including pay and working conditions.

I also hear often that in hospice care, there is a gap between what is advertised and what is actually provided. People may advertise that PT or OT visits the home, but the service may not be delivered sufficiently. Given that reality, I hope PACE will not stop at saying a service "is provided," but will ensure that it is actually delivered consistently in practice.

Thank you.

Caregiver 1

(omitted)

You must be very busy providing caregiving.
There is truly so much to keep track of. (omitted) After that, she continued to fall repeatedly. We lowered the bed as much as possible, but because her leg strength had weakened, it was hard for her to maintain balance. She fell again recently. I was told it was not severe enough to require surgery, but there was a fracture. At this point, she has almost no strength in her legs, so she has difficulty turning over on her own, standing, or even sitting up. So we have been going back and forth between the nursing facility and the emergency room. (omitted)

Could you tell me a bit about why your mother wanted to come back home while she was staying at the facility?
My mother did not have a serious chronic condition that required long-term placement in a facility. She was admitted temporarily for rehabilitation. But she contracted COVID-19 there, and her discharge was delayed. She kept saying she wanted to go home. In particular, she had strong complaints about the food. She used to cook well, so I think the facility meals did not suit her. Having to keep eating food that did not taste right seemed to cause her a lot of stress. Also, at that time, family visits were completely restricted. Later, we could contact her by video call about once a week, or if I visited the facility, we could only see each other through a glass window. Because we could not meet in person for so long, she kept asking why she had to stay there and repeatedly said she wanted to return home. Since she was emotionally very reliant on my father in daily life, she also often said she wanted to be with him. That desire was so strong that, in the end, I signed the discharge agreement because I wanted to honor her wishes. The medical staff advised me to think carefully, but I made the decision considering what my mother wanted. Because COVID had not ended yet, and second, they had seen through many patients how difficult it can be for families to care for an older adult at home. (omitted) I am not in very good health myself, and it felt too overwhelming for me to care for my mother at home, especially when she was still a patient. They were also very concerned, but I said I wanted to try as a final act of filial duty, so they connected us to hospice services and discharged her home. The hospice team also came and helped diligently, but I think the psychological stability she felt from having her wish fulfilled, that she was finally home, played a very important role.

(omitted)

At the time, I remember she received about 180 hours per month through IHSS. Two caregivers rotated, providing daytime care for five to six hours, and sometimes up to eight hours, depending on her condition. Those caregivers continued to care for her until fairly recently. My siblings and I also helped and kept monitoring her condition. As a result, at one point her health improved so noticeably that it was hard to believe. She seemed to return to a relatively stable older-adult life. So I thought we could finally feel a bit reassured, but then we faced another difficult situation.

(omitted)

Listening to you, it sounds like even though hospitals provide professional treatment and monitoring, they may not be able to attend to emotional needs as closely as family caregivers can.
That is unavoidable, right? They have so many people to care for.

So you have been accepting that reality and managing within it.
I complain when I think it is necessary, and I also check things directly. Even today, as soon as I got to the hospital, the first things I asked were how much my mother ate for breakfast, what her blood pressure was, and what her blood glucose levels were. From the providers' perspective, it could feel bothersome. They might think the family caregiver is overly strict. But over the past X years of caring for my mother, I have maintained a kind of daily log. When the caregiver arrives at 7 a.m., they measure and record fasting blood sugar and blood pressure. After she eats, we check her postprandial blood sugar again two hours later. I have tracked her status like that every day.

As her diabetes worsened, I recorded everything: when she received insulin, when she took vitamins, whether she rode a stationary bike for about ten minutes, and whether she went outside in her wheelchair for a 10–15 minute walk to get sunlight. I also asked the caregiver to keep a fairly detailed care log.

(omitted)

Before switching providers, was it difficult to change her primary care doctor?
No, it was not difficult. Once I signed to switch to XX, the previous one was automatically canceled.

(omitted)

I heard that at first, a few physicians were at the center of the planning, and as time went on, the program became more concrete. In my memory, it did not seem like a simple day care center, but rather a multi-purpose system with multiple functions integrated. So I felt it could become a meaningful model in the Korean community in terms of quality.

How did you feel when you first heard about it?
At first, I did not really understand what PACE was. I heard that it was already operating in other

communities, but not yet in Koreatown. So I felt positive about the idea that a model that is working well elsewhere would also be established in our community. I also had the impression that information was not being shared enough among Korean seniors. When my mother's health issues first began, I remember feeling completely lost about where to go and who to ask. There were hardly any official materials or brochures, and most of the time I had to rely on word of mouth. Even though the community has developed in many ways, access to information about integrated systems like this still felt limited. If a program like this were to operate fully, I thought I would want to actively tell people around me.

Recently, even seniors use YouTube a lot. If there were content explaining the purpose of the program, the benefits, eligibility, and how to contact them, simply sharing the link could have a big diffusion effect. I felt that if information were provided in a way that seniors could search for and understand on their own, it could meaningfully improve access and awareness.

At this point, is it true that most people still do not know about it?
Most people do not know.

Where do you usually get information?
I learned about it because staff explained it to me, but when I mentioned PACE to seniors in their 80s and 90s at church, most of them reacted as if it was the first time they had heard of it. Some were already using services like senior day care, and they tended to understand PACE as something similar. I explained that traditional day care is more like a partial service, whereas PACE integrates a wider range of supports, but many said they had never heard anything like that. My impression was that awareness is still very low.

When you first heard about PACE, did you ever think, "I would like my mom to go there and receive these benefits"?
Of course. (omitted) They said, legally, they can provide rides within about 20 miles or something like that. So I checked using my mom's ZIP code, and it was 20.5 miles.

What part did you look forward to the most?
I think what matters is that it is not just day care where someone stays for a few hours during the day, but a comprehensive, total-care model. If multiple programs are run together, there could be classes or activities that are genuinely helpful to seniors. When I look around, there are people who do not show it outwardly but seem to be struggling with depression. They live alone, hardly talk to anyone, and you can sense exhaustion in their facial expressions and overall demeanor. If trained professionals could provide enough time for conversation and activities under structured guidance, it would be very meaningful. One of the biggest concerns for seniors, I think, is how to spend their time in a meaningful and enjoyable way. People who would use a program like PACE are often not those with severe cognitive impairment, but those who can walk, or can use a wheelchair, and are still able to communicate. For them, it is important to have programs that go beyond basic caregiving and include meaningful activities and social interaction. Ultimately, I think the core is support that improves quality of life. And I also remember hearing that they plan to provide one nutritious meal a day. From the family's perspective, that would be reassuring. When seniors live alone, they often just eat something simple, skip meals, or lose their appetite.

But if they go to a center, they can at least receive a balanced meal on those days, and many of them really like the experience of eating together with others.

(omitted)

Even if the age criterion is 65 and older, different stories also come up among peers. People in their mid-70s sometimes joke that if they go there, they would be considered “too young.” A senior at church said something similar. When I asked why they did not go, they said it felt stifling and frustrating. Because there were many people in their 80s and 90s, those who were relatively younger were sometimes asked to help with various things. At home, they felt they had become very old, but there, they were treated as the younger person. They appreciated it in a way, but also found it burdensome. Another point was that seeing many people who are in very poor health up close can feel psychologically discouraging or uncomfortable. In that environment, they sometimes felt it was better not to go. They said it could lead to worries like, “Maybe I will end up like that too.”

If joining PACE requires changing the primary care doctor, how burdensome do you think that would feel?
I think it could feel quite burdensome for many seniors. It is not easy to change a doctor you have been seeing for a long time. If there is no major dissatisfaction with current care, that is even more true. Another thought is about capacity. If the program can serve, say, up to 500 people, and one particular physician is already seeing 300 patients, then many people might want that physician as their primary doctor. Then existing patients might worry that appointments would become harder to get or wait times would increase. In some clinics in the area, people already experience long waits even with scheduled appointments. Sometimes you make an appointment months in advance and still wait over an hour. Sometimes multiple people are booked for the same time slot. If those experiences keep happening, even if the program seems positive, people may worry about the inconvenience of actually using it. So I think it is important to secure enough clinicians so that both access and quality of care can be maintained. Expansion is welcome, but satisfaction will depend on whether the quality and accessibility are sustained.

What if joining PACE changes how IHSS is used?
Really? I did not know that.

For example, if someone is receiving 180 hours of IHSS support, PACE may not be able to provide the same number of hours. If joining requires stopping IHSS, how would that feel?
If PACE could cover a similar number of hours to what IHSS provides, I would definitely choose PACE. It would give my mother opportunities for social activity, rather than staying home. My mother could not walk well, so she could no longer attend the day care she used to go to. She was mostly at home, seeing only family and caregivers, and her daily range of activity was extremely limited. But even when I took her out occasionally, just to see flowers or to roll around a shopping mall in a wheelchair, her mood improved so much. That made me feel that staying home all the time is not necessarily good for seniors. So if PACE could provide support at a level similar to IHSS hours, I would be willing to choose it considering the integrated care and social activities. But if IHSS provides 180 hours and PACE provides only about ten hours, then the family would have to cover the difference, and that would be far too burdensome, so it

would be difficult to choose. Right now, because caregivers are present for those hours, I am still working, so I can step out briefly to handle my responsibilities, coordinate with staff, and then return to my mother. If that support disappeared entirely, I cannot be a full-time caregiver next to my mother because I have not retired yet.

When my mother was not doing well, my daughter who lives far away, my sibling in Korea, and my son who lives nearby all made time to come and be with her. They took turns staying for a few days at a time. My mother was very happy because she usually only saw me, but then all her children came.

These days, we use video calls often. I connect her with family in Korea or children who live far away for about 30 minutes a day, and through that I also gauge her cognitive condition. When she is not doing well, she sometimes does not recognize the person. But on some days, as soon as she sees the screen, she calls the person by name and asks how they are doing. Those reactions help me infer what kind of day she is having. Over time, I developed my own way of checking her condition through these calls. My children, watching this caregiving process, sometimes ask what kind of care I would want when I reach that age. I have told them clearly that if I am not able to make decisions for myself, I do not want aggressive life-prolonging treatments. If there is little chance of regaining consciousness, I do not want excessive measures taken. My mother says similar things. Sometimes she jokes and asks why they have not taken her yet. Hearing that brings up many thoughts. Recently, I told the social worker that our family does not want invasive surgery or life-prolonging treatments. Six years ago, we went through major surgery and multiple interventions without fully understanding the situation, but after experiencing that once, I do not want to make the same choice again. Later, when I showed my mother photos from that time, she also said, "Why did you go that far to keep me alive?" I did not interpret that as regret about the decision itself, but more as a wish not to repeat that kind of life-prolonging treatment.

Did you experience language-related difficulties, especially in medical situations?
Yes, it was difficult. When my mother was admitted through the emergency room, I could understand most explanations, but when specific medication names or medical terms came up, it was hard to know what they meant. Medical terminology is very different from everyday English, so it felt frustrating. So I asked the medical team to write down the medication names and the treatment plan. When I received it in writing and translated it, I had never heard those medication names before, but at least I could understand what they were trying to do. In the emergency room, they said decisions had to be made within 30 minutes, so the providers likely did not have much time to explain fully. In that situation, having to make decisions based on unfamiliar terms was the hardest part. A few days later, I was able to get a more detailed explanation from a nurse who was on duty, and only then did I understand the context of the treatment. For example, the term "G-tube" itself is not difficult, but I did not know exactly what the procedure involved. I only understood after someone explained it with a diagram. Looking back, it was not so much that I could not speak English, but that I was not familiar with specialized medical terminology. Because unfamiliar terms were suddenly tied to major decisions, I felt panicked. I think if there had been 24-hour medical interpretation, I would have been less overwhelmed. Real-time interpretation would have helped me make decisions when it was hard to understand professional terms immediately.

Recently, when she was in another facility, I saw a Korean interpretation system being used. It was a device with a screen through which an interpreter explained things in Korean in real time. The provider spoke in English and the interpreter conveyed it in Korean. But what I noticed was that the interpretation was accurate, yet it did not sufficiently consider the patient's age or level of understanding. Because the interpreter used relatively formal and complex Korean and explained things at length, it was actually hard to understand. So the family caregiver had to rephrase it in simpler words, and only then could my mother understand the questions and respond. So while the existence of interpretation is important, if the language is not adjusted to match the patient's cognitive status or comprehension level, it may not be practically helpful.

Thank you.

Caregiver 2

What was everyday life like while you were caregiving for your father?
We went to the hospital very often. We had to go to multiple departments, including nephrology, cardiology, and even foot-related care. Most of the time my husband took him, and when my husband could not, I went with them. Even when it was just for blood tests, we always went together.

So because it was difficult for him to use English, you had to accompany him each time.
When he was only doing outpatient visits, it was about two or three times a month. Before he started dialysis, it was once every one to two weeks. After he started dialysis, he received dialysis three times a week, and we also continued outpatient visits. Beyond the language issue, there were also physical challenges. He could not drive, and his walking was unsteady, so someone always had to go with him. My husband and I barely had any personal time, and there were many times when we had to leave work in the middle of the day to go to the hospital. Whenever an appointment was scheduled, someone had to make time, so it was an ongoing burden, and I remember it being extremely difficult. Before my father started dialysis, it was relatively manageable. We only had to accompany him to the hospital once or twice a month, and since my husband usually took him, I could respond by postponing work or taking a day off. But after he started dialysis, everything changed. We had to go to the hospital regularly three times a week, and with outpatient visits on top of that, it became practically impossible to keep working. From that point on, I stopped working, and I am still on leave to this day.

Other than dialysis and going to the hospital, have you ever used any other services?
No. In my father's case, he did not like going to crowded places. We also did not really look into much. It is the same with my mother now. She has severe dementia. She does not recognize family members, and she uses diapers.

Do you go to the hospital regularly now?
Right now, we go to the hospital once every two months, and a visiting nurse comes once a week. Because the primary care doctor placed orders, OT and PT also came once a week for a period of time. But OT and PT stop once the set period ends, and extensions are determined based on the doctor's judgment. In the end, the scope of services is limited by what the primary care doctor orders.

What criteria determine whether you can receive those services in the first place?
The doctor evaluated her and prescribed them. What is disappointing is that the duration is too short. After about 20 sessions, it ends. We are not paying for it ourselves, so I am not sure if the government covers it. From what I understand, it may be covered by the government, and maybe it is expensive, so they do not provide it for long. But I am not completely sure.

Your mother came to the U.S. after living in Korea, so language must be even more difficult for her.
Because she has severe dementia, it is hard to have a conversation. Even in the early stages of dementia, she could not speak English at all, so it was difficult for her.

Do you prepare all meals and everything yourself?
Yes. I prepare meals. If she lies down all the time, her legs stiffen, so I have to keep her moving and exercising. I also try to listen to her while watching TV together. And every day we go out and walk around the house for about 30 minutes.

How did you find your mother's primary care doctor?
He was originally my father's primary care doctor. We were very satisfied at the time, so we have continued seeing him as my mother's primary care doctor. He also recommended a dementia specialist, so we are seeing that doctor as well, and we continue regular visits with the primary care doctor. It has been about two years now.

Is the doctor Korean?
He can speak Korean, but I think he is more comfortable in English.

How did you find him?
My husband searched everything on Google. My father went to XX hospital for 30 years, so we looked for someone appropriate within that system. We checked where they went to school and things like that. My father has had high cholesterol for 20 or 30 years, and when he was looking for care, there was a well-known nephrologist at XX, so that is where we started. No, the doctor was not Korean. My husband always went with him. He is very devoted, so he always accompanied and took care of everything. It is hard, but they are your parents, so you endure it. Religion also helps a lot. We pray every day and go to church every weekend. Caring for my mother has been hard, but prayer has helped me a lot.

Do you share personal stories with people at church?
There is someone in the same situation as me. She is caring for her mother while working. Because we relate to each other, we understand. People who are not in this situation cannot fully understand.

Do you share information about doctors or hospitals as well?
Not really. People already have their own routines and their hospitals are already set. I think we mostly just talk about how hard it is. She has a caregiver, so she does not recommend hospitals or anything like that.

You mentioned that you accompany them to the hospital because of language barriers. Beyond that, were there other major inconveniences or things that worked well?
When we took her to the hospital, other than the staff being kind, I am not sure. But the visiting nurse has been extremely helpful. When my mother had diarrhea and we could not figure out why, the nurse said it could happen if she eats raw vegetables. We stopped giving her raw vegetables, and it improved. When she has wounds, the nurse checks everything. They also place orders for wipes, gloves, and things we need. When we go to the hospital, we are so exhausted that we cannot do anything afterward, but the nurse comes to us, so it is much easier. Having OT and PT come once a week was also good. It was convenient because they came to the house. They would come and do massage for an hour, teach us things, and my mother seemed to enjoy it. She also liked it because someone new was coming.

Are there aspects of caregiving that feel insufficient, inconvenient, or difficult?
There are so many inconveniences. I have to prepare all the meals and feed her. I have to bathe her too. It is hard that my husband and I have to spend so much time on this. And even though we only go to the hospital once every two months, it takes almost the entire day.

Have you ever tried looking for services like this?
Yes. We found a caregiver who comes four hours a day. My husband and I realized we simply could not do it alone.

(omitted)

Have you heard of PACE?
I am not sure. I guess it is a program for older adults.

It is a program that integrates medical and social services in one place, and it also includes transportation support. Have you heard of it?
No, I have not heard of it. Is it not the adult day health center? I see a lot of their vehicles driving around.

PACE, in Koreatown, is not available yet. There is a doctor at PACE. Now that you have heard about PACE, what do you think might be good about it?

If you go once, it seems like you could do things for a long time. After the appointment, you could also exercise. Maybe they would take better care of my mother. If she stays home all the time, it is boring, so I think it would be good if she could go somewhere like an "adult kindergarten," try different activities, and come back. Since there is nothing like that in Koreatown, I think people do not know anyone who has tried it. There is no real opportunity to talk about it. My husband and I have talked before about how it would be nice if we could go to a center like that once or twice a week and get some help. But if there is a doctor at PACE, that sounds good.

When your father was relatively healthy, did he ever go to a center like that?
No. He just said he did not want to. He did not like going to places where it was all elderly

people. He said it made him feel like he was too old, and he thought the atmosphere was too depressing. I do not think he felt lonely. He originally liked being alone.

If there were a PACE program in Korean for Korean older adults, what do you think?
I think it would be very good. They are older, and they cannot speak English. I think I would feel more comfortable sending her there. Even though my mother has dementia, when people speak to her in English, she does not respond. I think she recognizes it as a foreign language. It seems uncomfortable for her not to understand.

In what way would it feel more comfortable to send her to a place where Korean is available?
I think it is psychological comfort. Just knowing the language is shared makes it feel like she would be more at ease.

Do you have any concerns?
First, I would want to know the doctor's background and experience, and how long they have worked in this field. In my mother's case, she needs diaper changes, so I wonder if that kind of care is available. With severe dementia, she could have a bowel movement during activities, so I wonder if they can handle that type of care.

How do you feel about having to change your current primary care doctor?
We are already close with our current primary care doctor, so if we have to change doctors, we would have to think about it. He provides good care. When my father first went to a clinic in Koreatown, we did not realize it, but after we switched, this primary care doctor responded very quickly. But if the PACE doctor is very good, then we would consider it.

How did you end up going to a Korean doctor in Koreatown?
At that time, I think we searched online and went to a place that was known to have relatively good reviews. But it did not feel 100% certain, and it felt somewhat uneasy to fully trust them. The doctor also recommended that we go to a larger hospital, and we decided that an area like XXX, with many hospitals and a larger system, would be better than staying with a clinic in Koreatown, so we eventually moved.

Even though the Koreatown doctor was closer, you still switched?
Yes, we switched for my father's health. Since my husband took him anyway, my husband could handle the English, so the language issue was manageable. Even though it was farther and took more time, we moved for my father's health.

How was it different from doctors in Koreatown?
Sometimes it felt like the care in Koreatown was rushed. Even after the appointment, the explanations were not satisfying. With XX, I simply trust them. The process is well organized, and the doctor explains things extremely well. They talk in detail for 20 to 30 minutes, almost like family. Of course, we had to wait a lot and it was hard to get appointments, but once we were established with this primary care setting, it has been really good. They order tests appropriately and help us find the right testing locations. Even when we go together as a family, they take care of things carefully, like we are their own family, and it felt moving.

Would Korean-language services in PACE be a major advantage?
Yes. If the doctor at PACE is Korean, I think it would feel psychologically comfortable. My mother already has dementia, and it is not an acute or sudden illness, so I think a place that feels comfortable and that she can keep going to consistently would be better.

How do you think people around you would respond to a program like PACE?
I think they would respond positively. Just like I immediately thought it sounded good. There is no place where you can see a doctor and also do group activities. And since it is Koreatown, many older adults have difficulty with English, so I think it would feel psychologically comforting. Even considering the primary care doctor issue, it could still be a good option.

If enrolling in PACE means IHSS services would stop, what do you think?
I think people who are using IHSS would not go to PACE. Without a caregiver, daily life is not possible. Unless PACE can provide a similar number of hours to IHSS, I do not think it would work. Everyone considers IHSS very important. Without a caregiver, it is extremely hard. I also wonder whether they would accept someone like my mother, who uses diapers and has severe dementia. I also wonder if dental care is included. It feels unclear. If they say someone like my mother would be difficult, then it may not work. Many people with dementia would likely be there, including many with severe dementia. I have learned some ways to manage now, but in the beginning it was incredibly hard. I often think that elder caregiving is not easy.

Thank you.

Focus Group 1

Do you know about PACE?
No, not at all. Is it like an adult day health center?

PACE has physicians on site, including doctors, traditional Korean medicine providers, nurses, social workers, and occupational therapists, among others.

Are they all Korean?

Yes. We wanted to talk about what might be beneficial and what might be challenging if this kind of PACE program were established. In what ways do you think it would be helpful if the providers were Korean?

Being able to talk things through would help. For older adults, having someone to talk to is really important. For people like us who live in isolation, we truly need someone we can talk to. Even if the doctor is excellent, they are always busy, so you cannot really talk about these things. If we wanted to use it, what qualifications would we need?

From what I have heard, the overall model has been quite successful, but it seems there has not been a PACE designed specifically for Koreans. Structurally, it seems like a combination of a day care center and a medical center. Because there are also activity coordinators as staff, it is a model where activity programs and medical services are provided together. For Korean older

adults, it could be understood as a day care plus medical center that reflects Korean language and culture.

It sounds comprehensive. I am really looking forward to it. It would also be good because it is close to home. So I would need to meet the primary care doctor.

Have you experienced any inconvenience when using hospitals?
No. Everyone treats us well. If there is anything to improve, it is that overall hospitals are kind and do their jobs well. But there are some places where the nurses are a bit unfriendly. I have heard people talking about that in passing.

How do you feel when you see a Korean-speaking doctor?
It is very comfortable.

As you have gotten older and used hospitals, are there any difficulties you would like to share?
Not really. In Koreatown, my doctor is Korean.

What if your doctor only spoke English?
Oh, that would be too difficult.

It sounds like, overall, you are satisfied. Are there any improvements you would suggest? Do you ever have to visit multiple hospitals?

My insurance is XX, and I need my doctor to issue an authorization before I can go to another hospital. That is very inconvenient. Some places accept it and some do not. If I tell my doctor, they connect me, but then I have to wait and go again. I cannot handle it alone. It is hard to figure out.

Do you make these decisions on your own, or do you discuss them with your children?
I talk with my doctor and manage it on my own.

I do it on my own.

My children are all busy, so it is not common for me to ask them.

That is how it is for everyone. If it is a major surgery, they come with me, but for smaller things, I handle it alone. I can manage the smaller things. Also, the nurses are very kind, so I have not really felt inconvenience. They treat older adults well. So American hospitals are also kind. Korean providers are also kind, and I am very grateful. Living here feels so fortunate. They are so kind that I almost feel sorry.

Have you ever felt inconvenience when you needed to use English or handle something online?
I leave that to my kids. Computers.

So it seems computers are somewhat difficult for you.

Yes. I do not often need to use a computer, but when I do, I tell my kids to do it.

What kinds of activities do you do during the daytime?
I do not go to a center. I go to church and mostly exercise. I am not affiliated with any particular place.

I have diabetes, and these days I feel like I might be developing dementia. I have had diabetes for a long time, so I take so many medications. There are injections and pills, and it is exhausting. The daily ones are fine, but I was given a special medication device with specific instructions, and I do not know how to find it or how to use it. I went to the pharmacy and asked nurses, but they did not know. YouTube is in English, so I cannot understand it. I still do not know. It has been months, but I have not been able to use it. It is small, so even when I look at it, I cannot figure it out. They need to teach it more clearly. Even when they teach me, I forget quickly, and even when I try, it does not open. I cannot open it. It seems easy, but it is not. You do not know unless you have gone through it.

It sounds like someone needs to sit next to you and guide you step by step.

But it is hard to make appointments multiple times for that. The doctor just shows it quickly, but once I get home, it does not work.

Even when you go to the hospital, you only meet the doctor briefly.
Yes. Even if they teach me, I forget quickly. Even after seeing the person next door for years, it is hard to remember.

Your wife could help you.

Even if I learn and try to help, it does not work. The person needs to do it themselves.

I cannot open it. It seems easy, but it is not.

Even if I do it and teach, it does not work.

It is not that I am incapable or not smart, but I cannot even remember faces.

You have been speaking openly about physical health. How do you cope with things like dementia or depression? Those are illnesses too, but they can be hard to understand.
I do not have depression. But why would I not? If I call my son, he tells me not to call. In Korean workplaces, people have time to drink coffee and such, but he tells me not to call. At first, I did not understand what that meant and I felt hurt. Even when I call normally, he is not kind. I was always stressed, but now I understand more. I just accept that the world is like that. And these days, I have forgotten the stress a bit. I have no friends. Before, I used to meet friends in the U.S. and eat together, but now it feels bothersome to pretend to know people, and even talking feels tiring. I can talk if I do, but now I feel I need to live by forgetting stress.

Do you tell your doctor those things?
Yes, I do. And I cannot speak English. Even after learning for more than 60 years, I still cannot. I even served in KATUSA and I used to be good at English, but now I cannot understand it. Every day I study English by watching YouTube. I understand about a third of it here and there. Then I listen, eat, get sleepy, and sleep. Then I go to the library and study.

Do you do all of this on your own?
I used to go to adult school. I went a lot, but these days I do not. It is exhausting.

Some people spend their time in different ways on their own.

There is no one to meet, and it is hard for people to go to each other. So everything feels bothersome, and I just go to the park alone and walk one lap. Before, one lap was quick, but now it takes the entire morning. Then I feel sad.

So you do not attend a center or adult day care during the day.

Even if I do, if I go to Korean places, there are many people who show off. That creates more stress. Rather than being understood in an ordinary way, there are more situations where I get hurt.

I am dealing with things like depression and dementia on my own because it is hard to be understood at the hospital. I was born in XX. I do not know how I got this old. I feel very sad.

As you get older, time passes faster.

It is very hard. I am in pain night and day.

When you go to the hospital, it does not end with one visit. You have to make appointments at other places too. What if everything were in one place, including doctors, nurses, activities, traditional Korean medicine, dental care, physical therapy, and a whole team, and they also provided rides from home?
That does not exist. There is an adult day health center. They do activities, games, meals, and they provide transportation.

But in this model, there is a doctor on site.

That sounds good.

And if the whole team also speaks Korean.

That would be very good.

In what ways would it be good?

It would be good in many ways. There would be more pathways to help with paperwork, counseling, and so on.

At senior centers, they even say they can check emails and handle things for you. Receiving that kind of help is very good. If there is also a medical team, it would be perfect.

Have you ever heard that something like this exists?
Yes. I do not know which center it is, but people who attend say they always get checked like by a nurse, and there are exercise, singing, and many options depending on what they want to do.

It sounds like an adult day care center. But what if you could always see a primary care doctor there?

That would be very good.

Is that like an expensive nursing home, with doctors on site? Some expensive places have that.

Have you heard of something called PACE?
PACE?

It is a U.S. government program. As you get older and your health gets worse, even if you do not want to, you might end up going to a nursing home so that you do not become a burden to your children.

In American nursing homes, four people lie in beds and do not do activities. In Korea, there are luxury places. In the U.S., I have not heard of those. They might exist, but they would be extremely expensive, so ordinary people cannot even consider them.

Living like that and facing death is… it would be better to die of a heart attack.

From the government's perspective, nursing home costs are also very high. It is a major expense. So it is better for older adults, families, and the government if older adults can stay in the community and maintain their health. PACE was created for that purpose, and there are programs in multiple places across the U.S.

Is there one in LA?

Yes. There is. In LA too. You did not know?

No, I did not know.

It is a program that helps you keep living at home without entering a nursing home. But if you go, there will be many different ethnic groups. Even if you like the program a lot, would you go?

Because of language, I would not go.

You gain some sense over time, but I would not go to an English-speaking PACE.

If PACE were run by a Korean doctor and a Korean team, what would you think?

That would be good.

There would be no reason to dislike it.

But realistically, it does not exist.

I would have to live a long time to be able to use it.

For older people, if there is a place to rely on, it is very good.

PACE centers exist nationwide, and the LA area has many Asian residents, but if a PACE program were created for Koreans, a Korean-speaking medical team would meet and coordinate care. The team would make schedules, provide transportation, meals, and activities, and you could see a doctor.

What does "PACE" mean?

It is an integrated program or center for older adults.

That is good.

Because there would be a Korean-speaking team.

Older adults would not spend all day feeling depressed.

They would help with everything.

They would even read letters for you.

It was very successful in the Black community. But it did not exist in Koreatown. That is why we are doing interviews to help it take root successfully.

Please add one more thing. Educational components.

You need to make "PACE" entirely in Korean, with a term that immediately registers for anyone.

So the name does not feel intuitive.

For us, we are hearing it for the first time. It needs to become known.

If we do not know, we have to search on Google.

There should be a Korean term, like "nursing home," that conveys it.

If we think realistically, what would be an efficient approach for people to access PACE?
If it opens, we would have to come, right? Then do we have to pay anything?

No. It might seem very expensive, but you can receive those services. You need to have both Medicare and Medicaid, live in this area, and a doctor would evaluate your health status and sign that PACE is appropriate for you.

If we think broadly, it is a very good system, but if too many people want it, how can it handle that?

I understand each team has 12 people, and as the number of participants increases, the number of teams also increases.

Word spreads quickly. If people rush in like clouds, how do you handle that?

When the number of participants increases, the medical team increases according to government guidelines, based on ratios. And if you want to use PACE, you have to change your primary care doctor.

That is a difficult part, so you need to do good outreach. You need to explain how someone can get in.

Rather than the psychological burden of changing doctors, if people know how to change doctors, that is enough. You need to create a clear roadmap.

Even if you hear about it, you do not know who to ask or how to do it. So outreach has to be done well. You need to educate the people who receive the benefits. Those people can then share information with others they meet, and that makes it easier. But if only one person knows, then it is useless. Word of mouth spreads the fastest.

If I want to attend, do I have to change my doctor? That is not an easy decision. If I need it, I could change, but if I change hospitals, my information has to move too, so I cannot decide confidently. It feels hard to leave my current doctor.

It would feel inconvenient to have to get permission from my current doctor before switching.

For surgery, you have to wait for the primary doctor's authorization, so that is inconvenient.

You can change insurance for that.

I was wondering whether changing doctors feels burdensome or if it causes conflict.

Yes. If I need it, I could change. If I want to go somewhere better, then of course I would have to change. But whether I need to change insurance too, that is another issue.

First, having to change doctors.

I only have PPO, so I wonder if it works.

We have never changed from PPO. But we have Medicare and Medicaid…

Yes, you shared many points. It sounds like we would need a more intuitive name, and accurate information about eligibility requirements.

It would be so nice if everything could be handled neatly. But because of English, we cannot do it well. Even if we ask our kids, they do not know much about older-adult services. And if we ask someone we know, it takes a lot of time.

Thank you.

Focus Group 2
(omitted)

You were very curious about what PACE is. You can think of it as a comprehensive program for older adults. What do you think about the name?
I thought it was a cosmetics company.
I thought it meant going somewhere to exercise.
Never. From what I can tell, it seemed like it was close to XX.

As I mentioned earlier, this is a comprehensive model. It is structured so that various types of care are provided together within one building. There is an on-site primary care physician, dental care is available, and there are counseling staff and social workers, as well as staff who run programs and activities. The key feature seems to be that, in a single visit, you can receive integrated services across multiple domains in one place. It felt like a major advantage in terms of convenience and coordination because you do not have to visit multiple agencies separately.

What are the eligibility requirements to get in?
From my perspective, people say there is a PACE program, but hardly anyone even knows it exists. If people do not even know it exists, how are they supposed to use it? It does not make sense. Ask a few people, and none of them know.
So if no one knows about it, how can anyone use it? They do not even know it exists. There is no outreach at all. The basics are not in place.
It exists, but you do not even know it exists? That makes no sense. So the basics are not in place, in my view.
Like you said, there is no advertising. Even if we talk among ourselves, we will only recognize it briefly, and once we go home, we will forget that something like this exists. But if they keep promoting it consistently through Radio Korea, JoongAng Ilbo, or Korea Times, people would think, “Oh, something like this exists, maybe I should go check it out.”
The reason it is not in Koreatown is because people are not interested in things that do not make money. Everything is run for profit. It is excessive. It becomes a problem because the profit motive is excessive. If people hear there are benefits, that there are many benefits, they go

looking for it on their own. But it is not known, and if you do not even know what it is, how can you go look for it?
That has to come first. If you explain what is good about coming here, what is good about it—then it becomes beneficial for the person—then people will come on their own. If you cannot promote it publicly, it will die out. They have to find a way, somehow, to do what they can.
If you do not even know it exists, how can you use it?
I heard it for the first time today.
I thought it was a civic organization.
Do we have to get rid of all of our existing insurance?

If you are connected to the program, you will be guided and supported in overall aspects. Basically, it is for people aged 55 and older who have both Medicare and Medicaid, who live within the service area. Also, participation requires a referral through a primary care physician. Because there is an on-site primary care doctor at the center, I understand that enrollment proceeds through the referral process from the person's existing primary care physician.

I think people need to understand clearly how PACE is better than existing services in order to feel willing to switch their primary care physician and try it. But if that information is not repeatedly delivered through radio or television in everyday life, then when a problem arises, it is hard to recall where to go. You would need to have encountered it before to think, "I should contact that place," write down the phone number, and try.

If active outreach is difficult, it seems like it will be hard to make this service widely known. Word of mouth clearly has limits. If it is not sufficiently publicized even after the program exists, access could become even more difficult. It actually seems like a very useful service, so it feels unfortunate that its value is not being communicated adequately.

Among people who have difficulty using hospitals, many would probably want to use a system like PACE. Especially because medical costs are burdensome, a coordinated support structure could draw strong interest. If these advantages were well-known, demand would likely grow. But within the current system, it does not seem that information dissemination or access is happening sufficiently.

Also, because service availability depends on where you live, it feels like a limitation that participation is difficult if you do not live in the designated area.

I am curious about how many miles within the service area you need to live, what the income criteria are, and what the specific eligibility requirements are.

From what I can see, many Korean older adults use existing adult day health centers. But almost no one seems to know that PACE is beneficial. If, within adult day health centers, people talked about "PACE is good," those who know would naturally share it with others.

The problem is that because information about PACE itself is not delivered sufficiently, it is not clear who would introduce it first or who can actually connect people to it.

As I said earlier, the scope of social networks seems relatively narrow. Among Korean older adults, it is common for close relationships to be limited to two or three people, and information circulates within that structure. One person shares what they know with two or three close contacts, and it expands gradually through those contacts' acquaintances. So once an idea that a service is good is formed, it can spread quickly within a given network, but it is not easy for it to diffuse broadly beyond that network.

This is not about whether it is right or wrong. It is simply how things tend to work in practice. For example, in churches too, people often attend with those they are close to, and connections happen within those relationships. Because the main people one interacts with are relatively fixed, information diffusion also tends to occur in limited ways within that network.

If you meet eligibility criteria for the PACE program, one of the first concerns might be that you would need to change your primary care physician. How do you feel about changing your doctor?
If someone is satisfied with their current primary care physician, they may feel there is no need to switch. Of course, it depends on the person. If someone is dissatisfied with their current care, they might consider other options.

In my view, the competency of the primary care physician who would be working here would be a very important factor. Trust and willingness to participate could vary depending on how capable the physician leading PACE is. If the physician is well-known or has a strong reputation for treating a particular condition, it might naturally spread through word of mouth. Ultimately, the medical team's expertise and leadership could play an important role in building trust in the program.

At the same time, existing primary care physicians often operate private practices and maintain their own patient base. I also wonder whether the PACE physician would be working within a system funded by government financing. If the revenue structure is different, there could be concern that motivations or operational approaches might differ. I am not saying that is necessarily the case, but I can see how that question might arise.

And most importantly, people already have information about their current physician and have built trust through experience. Deciding to switch without knowing enough about the new physician could feel burdensome. Letting go of established trust and making a new choice may be perceived as involving a certain level of risk.

You mentioned trust. It seems the trust built over time with the primary care physician—knowing one's health history and having an established relationship—feels very significant.

Yes. Compared with a physician who operates a private practice, there may be a perception that a physician here has less autonomy or a different revenue structure, and that could raise concern that responsibility or focus might be lower.
I understand this not as saying it is true, but as saying such questions can naturally arise.

My current primary care physician has known my daily life and medical history for a long time, and because we have continued care for decades, trust has formed. So there is also an expectation that the physician will respond more quickly when needed. In contrast, with a new physician here, I would not know the specialty or expertise well, so it feels difficult to switch easily.

I was born in 19XX, and as I get older, I think it will become increasingly difficult to go from one hospital to another on my own. Right now, I still have a primary care physician and can get to appointments, but when my body gets weaker and mobility becomes harder, I thought a comprehensive system like this could be helpful. Especially if my health deteriorates significantly, it would feel reassuring to have a structure where overall management happens in one place.

I also think it could be an alternative that is different from a nursing home. But in the Korean community, because the immigration history is shorter than in some other communities and the population size is relatively smaller, it is possible that information diffusion and outreach have not been sufficient. Over time, it may become better known.

Ultimately, while I can still go to hospitals myself for now, as it becomes harder to move around or burdensome to go back and forth among multiple agencies, being referred into and using a coordinated system like this could become one viable option.

It also feels burdensome to ask one's children, and here everything is integrated in one place.

There are individual differences, and some people will go seek out places once they learn what is good. But when it becomes difficult to move around like that, having everything handled through one place by referral would be very convenient and helpful.

Also, if you have been seeing the same primary care physician consistently for eight or nine years or more, you become used to it and it feels comfortable. They also adjust prescriptions while knowing your situation.

I also have a primary care physician, but I am not always 100 percent satisfied. Even if there are inconveniences, I have been going for over ten years, so I just keep going. Each time I go to the hospital, I see residents, and I have rarely met the same physician repeatedly. That is inconvenient, but I have adapted. One reason I have not switched is that when another health issue arises, even if it takes time, they refer me to what I need—physical therapy, acupuncture, massage, and so on. I have received various benefits through that process.

But because there is not enough information about what is better than the current system, it seems hard to switch easily. In immigrant life, people often endure a certain level of inconvenience. In the end, it may also be that people do not switch because they do not know enough.

Thank you for sharing your thoughts today.
